# Lipid profile and safety of rosuvastatin monotherapy versus rosuvastatin plus ezetimibe in high risk coronary artery disease: a systematic review and meta-analysis of randomized controlled trials

**DOI:** 10.1186/s43044-025-00654-y

**Published:** 2025-06-10

**Authors:** Arga Setyo Adji, Atiyatum Billah, Angga Nugraha, Juliardi Eka Putra Sit, Bryan Gervais de Liyis, Abdillah Maulana Satrio Aji, Ragil Nur Rosyid, Bambang Edi Suwito

**Affiliations:** 1 Faculty of Medicine, Hang Tuah University, Indonesia; 2 Department of Cardiology, Gadjah Mada University, Yogyakarta, Center Java, Indonesia; 3 Department of Cardiology, Gadjah Mada University, Yogyakarta, Center Java, Indonesia; 4 Faculty of Medicine, Udayana University, Denpasar, Bali, Indonesia; 5 Department of Cardiology, dr. Ramelan Navy Hospital, Surabaya, Indonesia; 6 Department of Anatomy and Histology, Universitas Nahdlatul Ulama Surabaya, Surabaya, Indonesia

**Keywords:** Rosuvastatin, Efficacy, Safety, Coronary artery disease, Ezetimibe

## Abstract

**Background:**

Combining lipid-lowering agents may enhance outcomes in patients with high-risk coronary artery disease. While rosuvastatin is known to reduce LDL-C and cardiovascular events, the additional benefit of ezetimibe remains under investigation. This meta-analysis evaluated the efficacy and safety of RSV combined with EZ compared to RSV monotherapy in high-risk coronary artery disease.

**Methods:**

A systematic review was conducted using PubMed, Scopus, and Google Scholar up to August 30, 2024. Data were analyzed using a random-effects model in Review Manager 5.4. Lipid profile and safety outcomes were assessed in accordance with PRISMA guidelines.

**Results:**

Combination therapy with rosuvastatin and ezetimibe significantly improved the lipid profile in high-risk coronary artery disease patients compared to rosuvastatin monotherapy, based on 11 studies with 1,963 subjects. Treatment with RSV plus EZ decreased total cholesterol by 0.50 units (SMD = -0.50; 95% CI: -0.80 to -0.19; p = 0.001), LDL-C by 0.57 units (SMD = -0.57; 95% CI: -0.80 to -0.33; p < 0.00001), and triglycerides by 0.85 units (SMD = -0.85; 95% CI: -1.81 to -0.11; p = 0.002). Meanwhile, HDL-C increased by 0.26 units (SMD = 0.26; 95% CI: 0.04 to 0.48; p = 0.02). RSV monotherapy showed a significant risk of elevated liver enzymes (RR 0.36; 95% CI 0.13–0.99; *p* = 0.05), while combination therapy increased the risk of myalgia (RR 2.17; 95% CI 1.04–4.54; *p* = 0.04) and gastrointestinal symptoms (RR 2.00; 95% CI 1.01–3.97; *p* = 0.05). No significant difference in angina pectoris was noted (RR 0.84; 95% CI: 0.39–1.80; *p* = 0.65).

**Conclusion:**

Combination therapy with RSV and EZ effectively improves lipid profiles in high-risk coronary artery disease patients, particularly in reducing total cholesterol, LDL-C, and triglycerides. However, it is associated with a higher risk of gastrointestinal symptoms and myalgia. In contrast, RSV monotherapy is linked to a greater risk of elevated liver enzymes but was also associated with increased HDL-C compared to the combination therapy of RSV + EZ.

**Supplementary Information:**

The online version contains supplementary material available at 10.1186/s43044-025-00654-y.

## Background

The development of coronary artery disease (CAD), which continues to be a major cause of morbidity and death globally, is significantly influenced by dyslipidemia. Because statin medication, especially rosuvastatin (RSV), has been shown to be effective in lowering low-density lipoprotein cholesterol (LDL-C) and lowering the risk of severe cardiovascular events, it has long been a key component of the care of CAD. However, many individuals still have residual cardiovascular risk even after taking statins, which emphasizes the necessity for other therapeutic options [1]. The addition of ezetimibe (EZ), an agent that inhibits cholesterol absorption in the intestine, to statin therapy has been investigated for its potential to further reduce lipid levels and cardiovascular risk, demonstrating promising results in certain patient populations [1, 2].

The safety and efficacy of combining rosuvastatin and ezetimibe in patients with high-risk coronary artery disease warrant careful consideration. This therapeutic strategy, which targets different pathways of cholesterol metabolism, offers the potential for additive or synergistic effects [3]. Evidence suggests that RSV + EZ therapy not only improves lipid profiles more effectively than rosuvastatin alone but may also reduce cardiovascular morbidity [3, 4]. However, careful evaluation of the safety profile is required, as combination therapy may introduce additional side effects. Reported adverse events, including gastrointestinal disturbances, myalgia, and elevated liver enzymes, may influence treatment adherence [3–5]. The use of ezetimibe alongside rosuvastatin offers enhanced lipid-lowering potential but necessitates vigilant monitoring of tolerability, especially with respect to hepatic safety and muscle-related symptoms [6–8]. The potential impact of combination therapy on inflammatory markers and endothelial function has also garnered interest. Dual therapy may improve vascular health by reducing inflammation and enhancing endothelial function, which is particularly relevant in CAD, where endothelial dysfunction is prevalent [7]. Additionally, dual therapy may be advantageous for patients with metabolic comorbidities, such as type 2 diabetes mellitus (T2DM), which is common among CAD patients and is associated with worse clinical outcomes [8]. In terms of risk factors, hypertension, diabetes, and obesity are prominent contributors to CAD, and effective lipid management remains a critical intervention strategy. Studies from both the USA and Europe have underscored the importance of managing traditional cardiovascular risk factors such as smoking, hypertension, and diabetes to mitigate the risks associated with CAD [9]. Rosuvastatin has been effective in controlling LDL-C levels; however, residual cardiovascular risk, often linked to elevated triglycerides and low HDL-C, necessitates a combination approach. [10] 

The safety profile of ezetimibe, when used in combination with rosuvastatin, has shown mixed results. Some studies indicate that ezetimibe is generally well tolerated, while others report an increased incidence of adverse events, particularly gastrointestinal issues and muscular symptoms [11]. The potential for hepatic enzyme elevation is also a concern with statin therapy, and the addition of ezetimibe necessitates careful monitoring [12]. The cardiovascular benefits of improved lipid management in CAD have been well documented, and recent subgroup analyses have suggested that patients with diabetes may particularly benefit from the addition of ezetimibe to statin therapy, potentially due to enhanced effects on cholesterol absorption [13]. Furthermore, for patients with high cardiovascular risk profiles, including those with systemic inflammation or metabolic syndrome, the dual therapy of RSV + EZ may offer substantial benefits beyond LDL-C reduction [14]. Nonetheless, there is a need for more conclusive evidence on the safety and effectiveness of this combination therapy, particularly in patients with complex comorbidities. Although some studies have suggested that RSV + EZ therapy leads to improved outcomes in lipid management and potentially reduces cardiovascular events, there are conflicting results concerning its impact on glycemic control and liver function, especially in those with diabetes [5]. Current evidence suggests that while eplerenone and other similar agents might not significantly impact glucose levels, concerns about spironolactone remain, further complicating the management strategy for patients requiring dual lipid-lowering therapy. [15]

Given the current lack of meta-analyses focusing on the dual therapy of RSV and EZ, it is critical to perform a systematic review to synthesize existing evidence. This systematic review and meta-analysis aimed to evaluate the safety and efficacy of RSV + EZ in patients with high-risk coronary artery disease, providing new insights into how this combination therapy could be effectively integrated into treatment protocols for cardiovascular risk reduction. [16]

## Methods

This systematic review adhered to the PRISMA guidelines [17]. The review procedure has been registered under CRD42024600531 with the International Prospective Register of Systematic Reviews (PROSPERO). 

### Eligibility criteria

Predefined inclusion and exclusion criteria were used to choose the studies that were part of this systematic review and meta-analysis. Research was needed to evaluate the safety and effectiveness of RSV monotherapy versus RSV and EZ combined in high-risk CAD patients. To report effectiveness outcomes, including lipid profile metrics like LDL-C, HDL-C, TG, and total cholesterol, eligible studies are required. For inclusion, safety outcomes such as gastrointestinal complaints, myalgia, angina pectoris, and liver enzyme levels (SGPT/SGOT) were also crucial. Only randomized controlled trials (RCTs) published in English were included to ensure methodological rigor. Exclusion criteria comprised studies that did not make a direct comparison between the combination therapy and RSV alone, or those lacking relevant outcome measures. Nonhuman studies were excluded to maintain clinical relevance to the CAD patient population. 

### Search strategy and selection of studies

From August 2024 onwards, an extensive search was conducted in multiple databases, including PubMed, ScienceDirect, Google Scholar, and Europe PMC. The search strategy employed Boolean operators"AND"and"OR"using the following key terms: “coronary heart disease”, “CHD”, “Coronary Artery Disease”, “CAD”, “rosuvastatin”, “high-intensity lipid”, “moderate high-intensity lipid”, “RSV”, “ezetimibe”, “EZ”, and combinations such as “rosuvastatin plus ezetimibe” and “rosuvastatin combination”. This comprehensive search aimed to capture all relevant studies addressing the specified research question. Additionally, references from the selected articles were reviewed to identify further studies for inclusion. The complete search strategy is provided in Table S1 of the supplementary file. 

### Data extraction

Following the identification of pertinent studies, selected investigators (A.S.A., A.B., J.E.P.S., B.G.L., and A.N.) meticulously completed the data extraction procedure using a pre-established data extraction form. Key study characteristics like author, year, study design, study periods, location (country), population comparisons (RSV + EZ vs. RSV), mean age (years), and mean ± SD baseline values for LDL-C, HDL-C, TG, and total cholesterol in both intervention and control groups were among the data that were extracted. There was additional documentation of reported side effects (n, descriptions). A cross-checking procedure was used, in which a second investigator independently examined the extracted data to guarantee its completeness and accuracy. This validation process aimed to ensure the reliability and robustness of the data for further analysis.

### Quality assessment

Using the seven-step method suggested by the Cochrane Collaboration, a thorough evaluation of potential bias was carried out using the Cochrane Risk of Bias (RoB) Tool. To evaluate the risk of bias in the included studies, important components such as participant blinding, allocation concealment, randomization processes, incomplete outcome data, selective reporting, and other potential biases were closely investigated. The quality assessment was conducted by A.S.A., A.B., A.P., and I.A.T.P.A with any disagreements resolved collaboratively through discussion to maintain objectivity and consistency in the evaluation process.

### Outcome measure

The analysis encompassed both efficacy and safety outcomes. Efficacy was evaluated based on lipid profile parameters, including LDL-C, HDL-C, TG, and total cholesterol levels. Safety endpoints involved assessments of liver enzyme levels (SGPT/SGOT), gastrointestinal symptoms, myalgia, and angina pectoris.

### Data synthesis and statistical analysis

For every outcome measure, meta-analysis was used to compute the standardized mean differences (SMDs), risk ratios (RRs), and 95% confidence intervals (CIs). The atherogenic profile, which included LDL-C, HDL-C, TG, and total cholesterol, was one of the main effectiveness outcomes. Angina pectoris, myalgia, gastrointestinal symptoms, and liver enzyme levels (SGPT/SGOT) were examined in order to assess safety outcomes. Additionally, the impact of different dosing regimens on lipid parameters was analyzed. The I2 statistic was employed to assess heterogeneity among the included studies. Sensitivity analyses were performed to evaluate the robustness of the findings. A significance level of p < 0.05 was set, and statistical analyses were conducted using Review Manager 5.4. [18]

## Results

### Study selection process and quality assessment

**Fig. 1:**
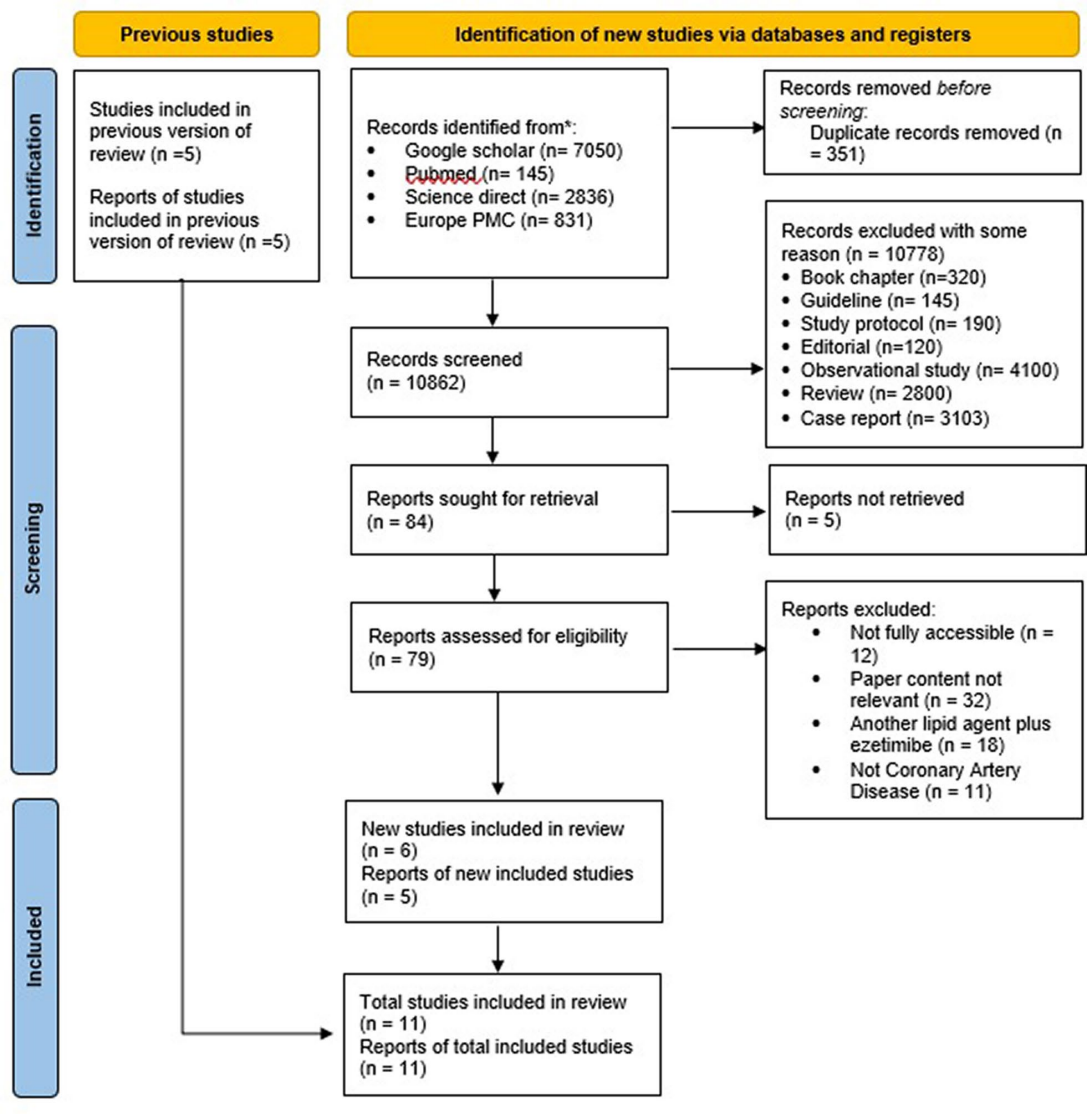
PRISMA flow diagram of the selection process

The search process identified a total of 10,862 records from multiple databases: Google Scholar (*n* = 7,050), PubMed (*n* = 145), ScienceDirect (*n* = 2,836), and Europe PMC (*n* = 831). After removing 351 duplicate entries, the remaining records underwent a rigorous screening process. A total of 10,778 records were excluded based on several factors: book chapters (*n* = 320), guidelines (*n* = 145), study protocols (*n* = 190), editorials (*n* = 120), observational studies (*n* = 4,100), reviews (*n* = 2,800), and case reports (*n* = 3,103). Out of the 84 reports retrieved for full-text screening, 5 were unavailable for retrieval. The remaining 79 reports were thoroughly reviewed, leading to the exclusion of 73 studies for reasons including inaccessibility (*n* = 12), irrelevance of content (*n* = 32), use of other lipid-lowering agents combined with EZ (*n* = 18), and lack of direct relevance to coronary artery disease (*n* = 11). Consequently, 6 new studies were included, resulting in a total of 11 studies being incorporated into the final analysis. A PRISMA flowchart, provided in Fig. 1, summarizes this selection process. ​

### Study characteristics

Table 1 provides a summary of the features of the included studies. The safety and effectiveness of combination therapy with RSV and EZ in patients with CAD are evaluated by the AGNEZS-E 2024 research, a systematic review and meta-analysis. 1, 3, 5, 19, and 22 Data from 11 studies carried out in four different countries—China (4 studies), Korea (2 studies), Japan (2 studies), and India (1 study)—are included in the review. Follow-up lasted anywhere from six to sixteen weeks. This multicenter investigation offers new insights into the role of RSV + EZ combination therapy in CAD management, particularly focusing on populations across Asia.

**Table 1:** Data characteristics

No	Author, year	Study design	Study periods	Location (country)	Population		Mean age (years)	Mean, SD baseline LDL levels in intervention group (mg/dl)	Mean, SD baseline HDL levels in control group (mg/dl)	Mean, SD baseline TG levels in intervention group (mg/dl)	Mean, SD baseline Total Cholesterol levels in intervention group (mg/dl)	Side effects (n, descriptions)
					RSV/EZ	RSV						
1	Ballantyne et al. 2007	RCT	2005–2006	Multicenter	239	230	63.5 ± 10.6 for RSV and 63.1 ± 10.2 for RSV plus EZ	R40 (191), R40 + EZ10 (189)	R40 (191), R40 + EZ10 (189)	R40 (186), R40 + EZ10 (186)	R40 (278), R40 + EZ10 (276)	Myalgia:—R (7), R + E (7) Nausea: R (5), R + E (6) Angina Pectoris: R (6), R + E (1) Abnormality value (AST or ALT > 3 × ULN): R (0), R + E (3) CK > 5 × ULN: R (2), R + E (2)
2	Bays et al. 2011	RCT	2009–2010	Multicenter	221	219	60.5 ± 10.0 for RSV (10 mg) and 60.5 ± 9.3 for RSV (5 mg) plus EZ (10 mg), 62.0 ± 9.1 for RSV (10 mg) plus EZ (10 mg), 61.4 ± 9.4 RSV (20 mg)	R5 + EZ10 (107 ± 23), R10 (102 ± 23), R10 + EZ10 (101 ± 27), R20 (98 ± 25)	R5 + EZ10 (52 ± 15), R10 (48 ± 12), R10 + EZ10 (54 ± 17), R20 (52 ± 13)	R5 + EZ10 (133 ± 80), R10 (143 ± 87), R10 + EZ10 (183 ± 32), R20 (178 ± 31)	R5 + EZ10 (188 ± 29), R10 (182 ± 29), R10 + EZ10 (183 ± 32), R20 (178 ± 31)	Abnormality value (AST or ALT ≥ 3 × ULN): R (0), R + E (1) CK ≥ 10 × ULN: R (1), R + E (0) CK ≥ 10 × ULN with muscle symptoms: R (0), R + E (0) Hepatitis-related: R (2), R + E (0) Gallbladder-related: R (0), R + E (0) GI-related: R (3), R + E (9) Allerhic reaction: R (1), R + E (3)
3	Choi et al. 2023	RCT	2018–2019	Korea	133	137	63.5 for RSV and 63.5 for RSV plus EZ	R20 (78.5), R10 + EZ10 (79.5)	NA	NA	NA	12 weeks: CK > ULN: R (13), R + E (7) CK > 4 × ULN: R (0), R + E (0) CK > 10 × ULN: R (0), R + E (0) AST or ALT > 4 × ULN: R (0), R + E (0) AST or ALT > 10 × ULN: R (0), R + E (0) 24 weeks:CK > ULN: R (16), R + E (10) CK > 4 × ULN: R (2), R + E (0) CK > 10 × ULN: R (0), R + E (0) AST or ALT > 4 × ULN: R (1), R + E (0) AST or ALT > 10 × ULN: R (1), R + E (0)
4	Joshi et al. 2017	RCT	2007–2008	India	80	80	59.78 ± 11.12 for RSV and 60.33 ± 9.83 for RSV plus EZ	R10 (153.38 ± 24.78),R10 + EZ10 (162.68 ± 23.13)	R10 (153.38 ± 24.78),R10 + EZ10 (162.68 ± 23.13)	R10 (39.38 ± 3.17),R10 + EZ10 (39.73 ± 2.56)	R10 (237.50 ± 25.67),R10 + EZ10 (247.13 ± 26.77)	Headache: - R (3), R + E (3) – > 12 wks—R (4), R + E (5) – > 24 wks Musculoskeletal side effects: - R (1), R + E (2) – > 12 wks—R (2), R + E (3) – > 24 wks GI side effects: - R (1), R + E (2) – > 12 wks—R (3), R + E (3) – > 24 wks
5	Masuda et al. 2015	RCT	2013–2014	Japan	21	19	70.2 for RSV and 64.0 for RSV plus EZ	R5 (123.0), R5 + EZ10 (131.8)	R5 (47.1), R5 + EZ10 (53.1)	R5 (144.9), R5 + EZ10 (129.7)	R5 (194.0), R5 + EZ10 (129.7)	MI: R (0), R + E (0) Death: R (0), R + E (0) Stroke: R (0), R + E (0) Rhabdomyolysis: R (0), R + E (0) Coronary revascularization: R (0), R + E (0) Abnormality value (AST or ALT > 3 × ULN): R (0), R + E (0) CK > 5 × ULN: R (1), R + E (1) Myalgia: R (1), R + E (0) Drug eruption: R (0), R + E (1) Discontinuation due to drug-related adverse event: R (1), R + E (1) Discontinuation due to other (renal failure): R (0), R + E (1)
6	Minyong et al. 2020	RCT	2017–2019	Korea	25	25	59.2 ± 9.7 for RSV and 59.27 ± 9.7 for RSV plus EZ	R20 (123.5 ± 32.8), R20 + EZ10 (127.5 ± 32.8)	R20 (45.0 ± 11.0), R20 + EZ10 (46.1 ± 13.9)	R20 (128.0 ± 78.7), R20 + EZ10 (121.0 ± 70.5)	R20 (183.0 ± 46.2), R20 + EZ10 (183.2 ± 33.2)	NA
7	Ran et al. 2017	RCT	2015–2016	China	42	83	60.6 ± 6.7 for RSV (10 mg), 60.5 ± 10.0 for RSV (20 mg), and 60.4 ± 8.2 RSV plus EZ	R10 (141 ± 33), R20 (141 ± 35), R10 + E10 (141 ± 27)	R10 (40 ± 9), R20 (40 ± 7), R10 + E10 (41 ± 6)	R10 (146 ± 31), R20 (146 ± 42), R10 + E10 (148 ± 42)	R10 (205 ± 37), R20 (208 ± 46), R10 + E10 (207 ± 32)	CK ≥ 5 × ULN: R (1), R + E (0) Muscle pain: R(6), R + E (2) Rash: R(1), R + E (0) GI discomfort: R(0), R + E (2) Rhabdomyolysis: R(0), R + E (0) AST or ALT > 3 × ULN: R (0), R + E (0)
8	Sun et al. 2021	RCT	2019–2020	China	81	90	64.08 ± 10.45 for RSV and 61.74 ± 8.78 for RSV plus EZ	R20 (113.69 ± 40.99), R20 + E10 (114.08 ± 32.87)	R20 (39.44 ± 10.44), R20 + E10 (42.15 ± 10.44)	R20 (183.29 ± 130.29), R20 + E10 (173.55 ± 122.19)	R20 (117.50 ± 53.36), R20 + E10 (175.95 ± 48.34)	CV death: R (3), R + E (1) Nonfatal MI: R (4), R + E (3) UA: R (10), R + E (8) Revascularization: R (7), R + E (5) Nonfatal stroke: R (0), R + E (0)
9	Wang et al. 2016	RCT	2011–2014	China	50	48	65 ± 12.0 for RSV and 63 ± 10.0 for RSV plus EZ	R10 (3.48 ± 1.26), R10 + E10 (3.62 ± 1.18)	R10 (1.13 ± 0.22), R10 + E10 (1.13 ± 0.21)	R10 (1.90 ± 0.65), R10 + E10 (1.97 ± 0.67)	R10 (5.58 ± 02.58), R10 + E10 (1.97 ± 0.67)	New MI: R (1), R + E (0) Recurrent MI: R (0), R + E (0) UAP:: R (5), R + E (2) Cardiac death: R (0), R + E (0) Stroke: R (0), R + E (0) Myalgia: R (1), R + E (1) Abnormality value (AST or ALT > 3 × ULN): R (1), R + E (2) CK > 5 × ULN: R (0), R + E (0) Rhabdomyolysis: R (0), R + E (0)
10	Xin et al. 2016	RCT	2011–2013	China	70	70	62.2 ± 9.7 for RSV and 64.3 ± 10.1 for RSV plus EZ	R10 (2.48 ± 0.66), R10 + E10 (2.52 ± 1.07)	R10 (1.13 ± 0.19), R10 + E10 (1.08 ± 0.24)	NA	R10 (4.28 ± 0.87), R10 + E10 (4.42 ± 1.32)	Abnormality value (AST or ALT > 3 × ULN): R (1), R + E (2) Rhabdomyolysis: R (0), R + E (0) Myalgia with CK > 5 × ULN: R (1), R + E (0) Cancer: R (0), R + E (0) Myopathy: R (0), R + E (0)
11	Yamazaki et al. 2013	RCT	2011–2012	Japan	22	24	71.8 ± 8.2 for RSV and 70.1 ± 9.6 for RSV plus EZ	R10 (88.5 ± 12.9), R2.5 + E10 (84.3 ± 14.5)	R10 (46.4 ± 11.6), R2.5 + E10 (49.9 ± 12.2)	R10 (165.4 ± 78.9), R2.5 + E10 (149.4 ± 103.9)	R10 (168.0 ± 17.4), R2.5 + E10 (164.0 ± 23.3)	NA

### Risk of bias

**Fig. 2:**
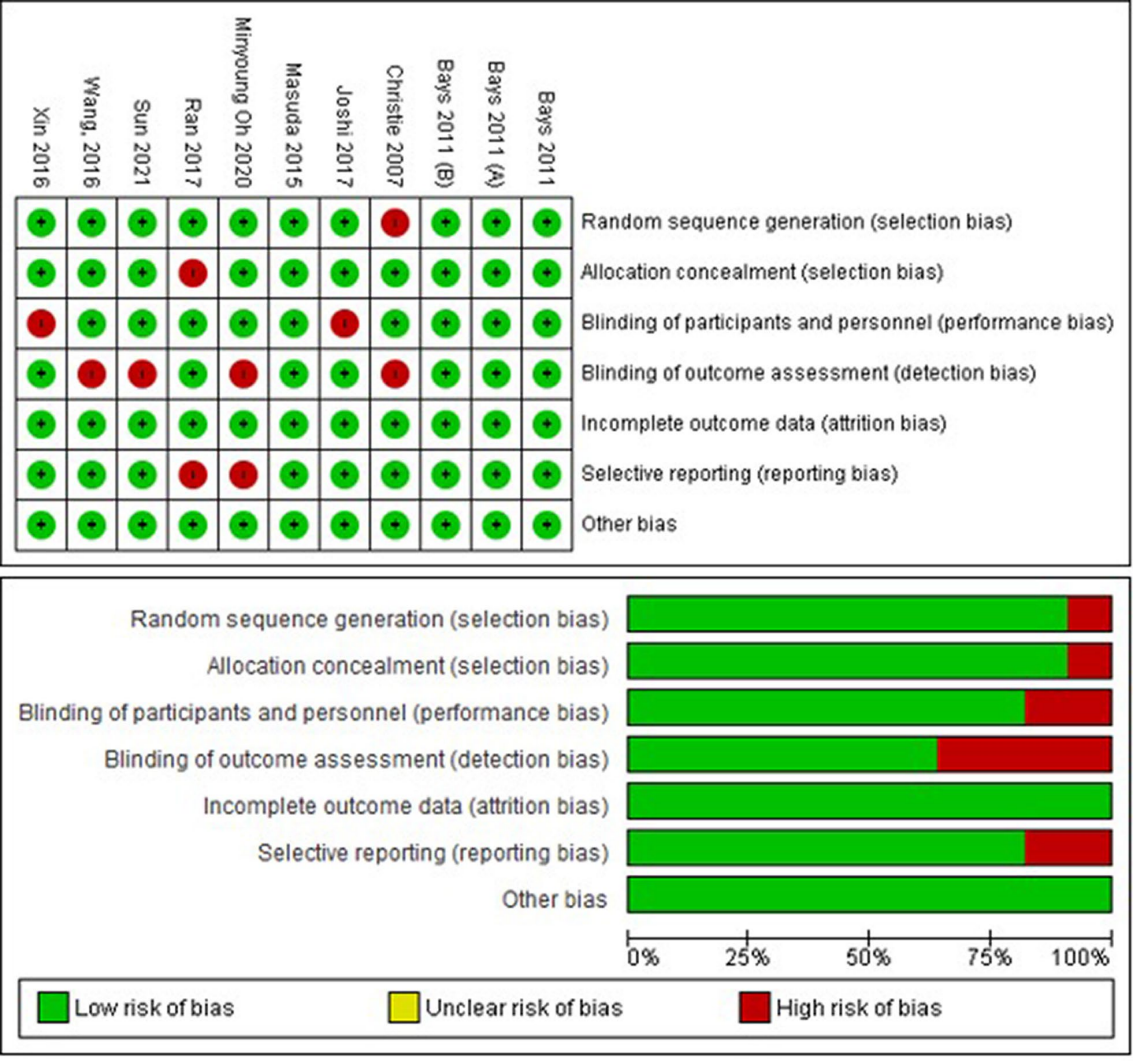
Risk of bias graph and summary

The 11 included studies were evaluated for risk of bias using the Cochrane RoB Tool, and the results are summarized in Fig. 2 and Table 2. All studies were assessed to have a low risk of bias, indicating a high level of methodological rigor across the investigations. This suggests that the data synthesized in this review are of high quality, providing reliable insights into the efficacy and safety of the RSV + EZ combination in CAD patients.

**Table 2:** Study outcomes

No	Author, year	Drug comparator	Treatment duration	Main result	Key outcomes	Risk of bias/quality of study
1	Ballantyne et al. 2007	I:RSV, C:RSV plus EZ	6 weeks	When given combination rosuvastatin 40 mg and ezetimibe 10 mg (*n* = 239) to individual with high risk of coronary heart disease, significantly achieved Adult Treatment Panel III (ATP III) with LDL-C goal < 100 mg (94%), while rosuvastatin monotherapy alone (*n* = 230) achieved 79,1%. This study also shown other component of the lipid/lipoprotein profile were also significantly improved with combination rosuvastatin and ezetimibe	Rosuvastatin combined with ezetimibe significantly improved LDL cholesterol levels in 94% of high-risk coronary heart disease patients, achieving better lipid goals than monotherapy, with no serious adverse events reported	Low
2	Bays et al. 2011	I:RSV, C:RSV plus EZ	6 weeks	Giving Ezetimibe 10 mg with RSV 5 mg or 10 mg, lowers LDL-C better by 21% compared to up-titration of RSV 10 mg or 20 mg which only lowers LDL-C by 5.7%. This combination of EZ and RSV also shows greater reductions in total cholesterol, non-high-density lipoprotein cholesterol, and apolipoprotein B (*p* < 0.001)	Ezetimibe added to rosuvastatin improved outcomes by achieving a 21% reduction in LDL cholesterol and higher LDL cholesterol target attainment in 59.4% of high-risk patients, compared to 30.9% with rosuvastatin up-titration	Low
3	Choi et al. 2023	I:RSV, C:RSV plus EZ	24 weeks	This study found that after 12 and 24 weeks of treatment, combination of Rosuvastatin 10 mg with Ezetimibe 10 mg show greater reduction in LDL-C level compared to monotherapy of rosuvastatin 20 mg. It has also greater number of patients achieved the target LDL-C level of ≤ 70 mg in combination group during treatment period (12–24 weeks, respectively). There were no AEs and ADs reactions between two groups	Combination therapy of moderate-intensity rosuvastatin and ezetimibe led to a greater reduction in LDL cholesterol levels, with a decrease of −22.9% after 12 weeks and −24.2% after 24 weeks, compared to high-intensity rosuvastatin monotherapy, which showed a decrease of −15.6% after 12 weeks and −12.9% after 24 weeks	Low
4	Joshi et al. 2017	I:RSV, C:RSV plus EZ	24 weeks	The impact on the lipid profile was computed following 12 and 24 weeks of treatment. After 12 weeks, the results indicated that Group II (rosuvastatin + ezetimibe) had considerably higher percentage decreases in LDL-C (53.65 vs 41.13), TGs (26.29 vs 18.39), and TC (38.98 vs 28.91), as well as higher elevations in HDL-C (9.56 vs 7.08), compared to Group I (rosuvastatin alone) (*p* < 0.01). Similarly, following 24 weeks of treatment, Group II experienced considerably greater increases in HDL-C (7.73 vs 5.09) and decreases in LDL-C (57.39 vs 45.54), TGs (30.68 vs 21.88), and TC (41.92 vs 32.16) than Group I (*p* < 0.01)	After 24 weeks of treatment, the combination of rosuvastatin and ezetimibe resulted in greater reductions in lipid parameters compared to rosuvastatin alone. LDL-C decreased by 57.39% versus 45.54%, triglycerides by 30.68% versus 21.88%, and total cholesterol by 41.92% versus 32.16%. Additionally, HDL-C increased by 7.73% with the combination therapy compared to 5.09% with rosuvastatin alone	Low
5	Masuda et al. 2015	I:RSV, C:RSV plus EZ	24 weeks	The combination group's LDL-C level was considerably lower (−55.8%) than the monotherapy group's (−36.8%; *P* = 0.004). The combination group seemed to reduce the primary endpoint, the percent change in plaque volume (PV), more successfully than the monotherapy group (−13.2% versus −3.1%, respectively, *P* = 0.050). Additionally, the effects of the two therapies on PV showed a significant group × time interaction (*P* = 0.021), suggesting that for slightly different baseline PV values in the two treatment groups, the combination therapy had a larger regressive effect on PV than monotherapy. Additionally, there was a positive association between the percent change in PV and the percent change in LDL-C (*r* = 0.384, *P* = 0.015)	The combination therapy with ezetimibe and a statin provided a significant reduction in small dense LDL (sd-LDL) of up to 46%	Low
6	Minyong et al. 2020	I:RSV, C:RSV plus EZ	24 weeks	Both treatment groups, ezetimibe/rosuvastatin and high-dose rosuvastatin, showed significant reductions in total cholesterol and LDL cholesterol levels after 6 months (*p* < 0.05). However, high-sensitivity C-reactive protein levels decreased significantly only in the ezetimibe/rosuvastatin group (*p* = 0.046), while triglyceride and HDL cholesterol levels did not change significantly in either group	After 6 months of treatment, both the ezetimibe/rosuvastatin combination and high-dose rosuvastatin alone significantly reduced total cholesterol and LDL cholesterol levels (*p* < 0.05). However, only the combination therapy significantly decreased high-sensitivity C-reactive protein levels (*p* = 0.046), while triglyceride and HDL cholesterol levels did not change significantly in either group	Low
7	Ran et al. 2017	I:RSV, C:RSV plus EZ	12 weeks	At week 12, rosuvastatin (20 mg) and rosuvastatin (10 mg) were significantly lower than the combination therapy group in the primary end point (81.0% vs. 68.3% vs. 33.3%, P b 0.001). Similarly, at week 12, there was a decrease in LDL-C levels (67.28% vs. 52.80% vs. 43.89%, *P* < 0.001). Compared to the rosuvastatin 10 mg group and the combination therapy group, the rosuvastatin 20 mg group experienced a significantly greater incidence of drug-related side events (17.0% vs 2.4% vs 4.8%, P b 0.05)	After 12 weeks of treatment, the combination of ezetimibe and rosuvastatin resulted in a lower proportion of cardiovascular events compared to rosuvastatin alone. The cardiovascular event rates were 4.8% for the combination therapy group, 11.9% for the rosuvastatin 10 mg group, and 9.8% for the rosuvastatin 20 mg group, although the differences were not statistically significant (*P* > 0.05)	Low
8	Sun et al. 2021	I:RSV, C:RSV plus EZ	1 weeks	Total cholesterol (TC) and low-density lipoprotein cholesterol (LDL-C) plasma levels were reduced more when ezetimibe and rosuvastatin were used together than when rosuvastatin was used alone. In particular, on day 7 following PCI, the combination group experienced a 14.1% decrease in TC compared to the rosuvastatin group's 5.9% decrease (*p* = 0.02), while the LDL-C group experienced a 15.6% decrease compared to a 6.1% decrease (*p* = 0.04). After seven days of treatment, there was no statistically significant change between the two groups'levels of triglycerides (TG) and high-density lipoprotein cholesterol (HDL-C)	Specifically, on day 7 after PCI, the reduction in TC was 14.1% in the combination group versus 5.9% in the rosuvastatin group (*p* = 0.02), and the reduction in LDL-C was 15.6% versus 6.1% (*p* = 0.04). High-density lipoprotein cholesterol (HDL-C) and triglyceride (TG) levels were not significantly reduced in either group after 7 days of treatment and showed no statistical difference between the two groups	Low
9	Wang et al. 2016	I:RSV, C:RSV plus EZ	48 weeks	At six and twelve months following treatment, ezetimibe + rosuvastatin reduced levels of MMP-9, hsCRP, IL-6, total cholesterol, and low-density lipoprotein cholesterol. A statistically significant difference between the two groups was found. Plaque load, plaque cross-sectional area, and necrotic plaque composition percentage were all significantly lower in the combination group at 12 months compared to the rosuvastatin alone group (*P* < 0.05). Additionally, the primary endpoint dropped more successfully in the combination group than in the rosuvastatin alone group	Ezetimibe and rosuvastatin resulted in a significant reduction in low-density lipoprotein cholesterol (LDL-C) levels compared to rosuvastatin monotherapy (*P* < 0.05)	Low
10	Xin et al. 2016	I:RSV, C:RSV plus EZ	15 weeks	LDL-C values were considerably lower in the Rosuva/Ez group (rosuvastatin plus ezetimibe) than in the Rosuvastatin monotherapy group. After 12 weeks, the Rosuva/Ez group's LDL-C level was considerably lower than the Rosuva group's (*P* < 0.001). Furthermore, during the course of the research, the dual lipid-lowering approach demonstrated a more significant decrease in LDL-C levels than rosuvastatin monotherapy (*P* < 0.001)	Specifically, at the end of 12 weeks, the LDL-C level was significantly lower in the Rosuva/Ez group compared to the Rosuva group (*P* < 0.001). The dual lipid-lowering strategy showed a more marked reduction of LDL-C level than rosuvastatin monotherapy throughout the study (*P* < 0.001)	Low
11	Yamazaki et al. 2013	I:RSV, C:RSV plus EZ	16 weeks	The groups'baseline characteristics did not differ appreciably. At 12 weeks, there was no significant difference between the two groups in LDL-C and inflammatory markers (hsCRP, interleukin-6, tumor necrosis factor-alpha, and pentraxin 3) (LDL-C: R10 vs. R2.5/E10: −19.4 ± 14.2 vs. −22.4 ± 14.3 mg/dL). In contrast to the R2.5/E10 group, the R10 group's high-density lipoprotein cholesterol (HDL-C) was considerably better (4.6 ± 5.9 vs. 0.0 ± 6.7 mg/dL; *p* < 0.05)	After 12 weeks, both treatments similarly reduced LDL-C, T-Cho, TG, LDL-C/HDL-C ratio, and MDA-LDL. However, HDL-C was significantly improved in the R10 group compared with the R2.5/E10 group, with no significant differences in changes in inflammatory markers between the two groups However, R10 elevated HDL-C more effectively than R2.5/E10	Low

Table

### Efficacy of rosuvastatin versus rosuvastatin plus ezetimibe

**Fig. 3:**
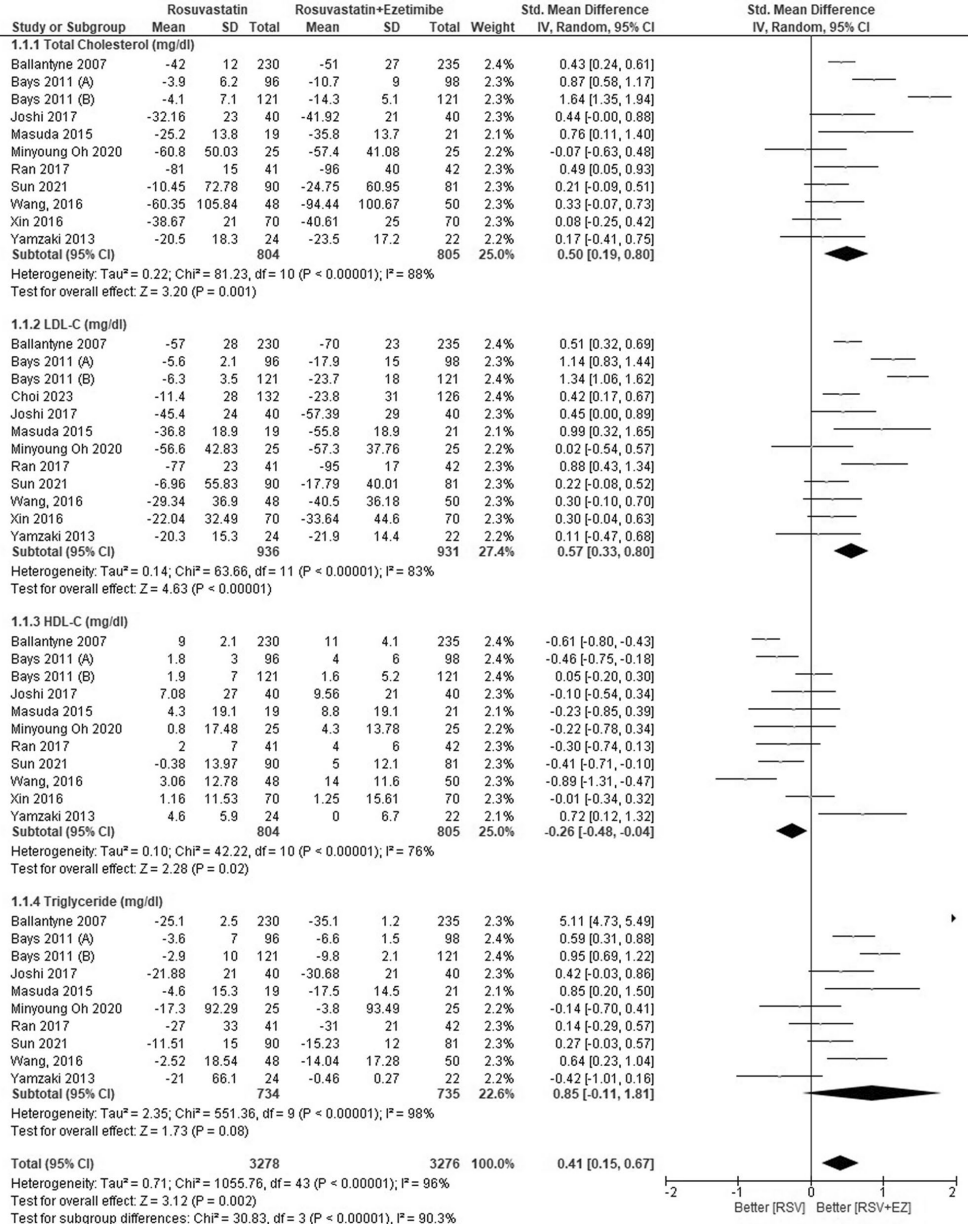
Efficacy outcome of RSV vs RSV plus ezetimibe on lipid profile (TC, LDL-C, HDL-C, TG)

The overall findings are presented in Table 3. The lipid profiles of patients with high-risk CAD were evaluated across eleven studies comparing RSV monotherapy to the combination therapy of RSV + EZ (Fig. 3). Significant decreases in the whole lipid profile, including triglycerides, LDL-C, and total cholesterol, were the outcome of the combination therapy, while RSV monotherapy was better at increasing HDL-C. In particular, RSV + EZ showed a substantial reduction in LDL-C levels (SMD = 0.57; 95% CI = 0.33—0.80; *p* < 0.00001) and total cholesterol levels (SMD = 0.50; 95% CI = 0.19–0.80; p = 0.001). Furthermore, there was a substantial increase in HDL-C levels (SMD = − 0.26; 95% CI = − 0.48 to − 0.04; *p* = 0.02) and triglycerides (SMD = 0.85; 95% CI = − 0.11 to − 1.81; *p* = 0.002). Overall, the combination of RSV with EZ provided substantial improvements in the lipid profiles of CAD patients compared to statin monotherapy.

**Table 3:** Summary of results

End Point	No. of studies	Standard Mean Difference/SMD [95% CI]	*P* values
*Efficacy*			
TC	10 study	SMD 0.50 [0.19–0.80]	0.001^*^
LDL-C	11 study	SMD 0.57 [0.33–0.80]	< 0.00001^*^
HDL-C	10 study	SMD −0.26 [−0.48- −0.04]	0.002^*^
TG	9 study	SMD 0.85 [−0.11–1.81]	0.008^*^

Safety	RSV/EZ	RSV	Risk Ratio/RR [95% CI]	*P* values
Liver enzyme (SGPT/SGOT)	2.87% (21/731)	0.83% (6/720)	RR 0.41 [0.15–1.11]	0.05^*^
Gastrointestinal symptoms	3.01% (11/366)	6.79% (25/368)	RR 2.00 [1.01–3.97]	0.05^*^
Myalgia	1.57% (10/637)	4.32% (29/671)	RR 2.05 [0.98–4.30]	0.04^*^
Angina pectoris	6.01% (26/432)	4.77% (23/482)	RR 0.49 [0.35–0.71]	0.65

Lipid profile subgroup analysis		Standard mean difference/SMD [95% CI]	*P* values
Dose intensity	No. of studies		
Low-Dose RSV (RSV/EZ 2.5–5 mg/10 mg vs RSV 5–10 mg)	4 study	TC: SMD 0.46 [−0.03–0.94]	0.007^*^
		LDL-C: SMD 0.58 [−0.04–1.20]	0.007^*^
		HDL-C: SMD −0.08 [−0.58–0.42]	0.75
		TG: SMD 0.23 [−0.32–0.78]	0.41
Moderate-Dose RSV (RSV/EZ 10 mg/10 mg vs RSV 10 mg)	6 study	TC: SMD 0.79 [0.17–1.41]	< 0.00001^*^
		LDL-C: SMD 0.85 [0.50–1.20]	< 0.00001^*^
		HDL-C: SMD −0.26 [−0.53–0.01]	0.003^*^
		TG: SMD 0.49 [0.16–0.82]	0.004^*^
High-Dose RSV (RSV/EZ 20–40 mg/10 mg vs RSV 20–40 mg)	2 study	TC: SMD 0.43 [0.24–0.61]	< 0.00001^*^
		LDL-C: SMD 0.48 [0.19–0.80]	< 0.00001^*^
		HDL-C: SMD −0.61 [−0.80- −0.43]	< 0.00001^*^
		TG: SMD 5.11 [4.73–5.49]	< 0.00001^*^

Treatment Duration	No. of Studies		*P* values
Short-Term (≤ 6 weeks)	6 study	TC: SMD 0.61 [0.21–1.01]	0.003^*^
		LDL-C: SMD 0.73 [0.36–1.10]	0.0001^*^
		HDL-C: SMD −0.31 [−0.60- −0.02]	0.04^*^
		TG: SMD 1.04 [−0.20–2.29]	0.10
Mid-Term (12 weeks)	5 study	TC: SMD 0.22 [−0.03–0.48]	0.08
		LDL-C: SMD 0.47 [0.26–0.68]	< 0.0001^*^
		HDL-C: SMD 0.03 [−0.32–0.38]	0.87
		TG: SMD 0.08 [−0.36–0.52]	0.72
Long-Term (> 12 weeks)	5 study	TC: SMD 0.35 [0.06–0.63]	0.002^*^
		LDL-C: SMD 0.40 [0.19–0.61]	0.0002^*^
		HDL-C: SMD −0.38 [−0.78–0.02]	0.06
		TG: SMD 0.44 [0.06–0.81]	0.02^*^

The lipid profile subgroup analysis by dose intensity is presented in Fig. 6. In the low-dose group (RSV/EZ 2.5–5 mg/10 mg vs RSV 5–10 mg), there was a reduction in total cholesterol (TC) (SMD 0.46; 95% CI [− 0.03, 0.94], *p* = 0.007) and LDL-C (SMD 0.58; 95% CI [− 0.04, 1.20], *p* = 0.007), no significant change in HDL-C was observed (SMD −0.08; 95% CI [− 0.58, 0.42], *p* = 0.75), and triglycerides showed only a reduction (SMD 0.23; 95% CI [− 0.32, 0.78], *p* = 0.41). In the moderate-dose group (RSV/EZ 10 mg/10 mg vs RSV 10 mg), significant improvements in lipid profiles were noted, with reductions in TC (SMD 0.79; 95% CI [0.17, 1.41], *p* < 0.00001), LDL-C (SMD 0.85; 95% CI [0.50, 1.20], *p* < 0.00001), and triglycerides (SMD 0.49; 95% CI [0.16, 0.82], *p* = 0.004). However, a slight increase in HDL-C was observed (SMD −0.26; 95% CI [− 0.53, 0.01], *p* = 0.003). Finally, in the high-dose group (RSV/EZ 20–40 mg/10 mg vs RSV 20–40 mg), significant reductions in TC (SMD 0.43; 95% CI [0.24, 0.61], *p* < 0.00001) and LDL-C (SMD 0.48; 95% CI [0.19, 0.80], p < 0.00001) were observed. However, HDL-C significantly increases (SMD −0.61; 95% CI [−0.80, −0.43], *p* < 0.00001), and triglycerides showed a substantial reduction (SMD 5.11; 95% CI [4.73, 5.49], *p* < 0.00001). Overall, the moderate- and high-dose RSV/EZ combination therapies showed significant lipid-lowering effects, particularly in reducing TC, LDL-C, and triglycerides, although higher doses were also associated with an increase in HDL-C.

The lipid profile subgroup analysis based on treatment duration is presented in Fig. 7. In the short-term treatment group (≤ 6 weeks), combination therapy of RSV + EZ showed significant improvements in total cholesterol (TC) (SMD 0.61; 95% CI [0.21, 1.01], *p* = 0.003) and LDL-C (SMD 0.73; 95% CI [0.36, 1.10], *p* = 0.0001), with a slight increase in HDL-C (SMD −0.31; 95% CI [− 0.60, − 0.02], *p* = 0.004), while triglycerides (TG) did not show a significant reduction (SMD 1.04; 95% CI [− 0.20, 2.29], p = 0.10). In the mid-term treatment group (12 weeks), while LDL-C showed a significant reduction (SMD 0.47; 95% CI [0.26, 0.68], *p* < 0.0001), TC did not show a significant change (SMD 0.22; 95% CI [− 0.03, 0.48], *p* = 0.08), and HDL-C and TG also showed no significant differences (SMD 0.03; 95% CI [− 0.32, 0.38], *p* = 0.87 for HDL-C, SMD 0.08; 95% CI [− 0.36, 0.52], *p* = 0.72 for TG). In the long-term treatment group (> 12 weeks), TC and LDL-C both showed significant reductions (SMD 0.35; 95% CI [0.06, 0.63], *p* = 0.002 for TC, SMD 0.40; 95% CI [0.19, 0.61], *p* = 0.0002 for LDL-C), while HDL-C showed a non-significant increase (SMD − 0.38; 95% CI [− 0.78, 0.02], *p* = 0.06). TG showed a significant reduction in the long-term group (SMD 0.44; 95% CI [0.06, 0.81], *p* = 0.02). Overall, the results indicate that the combination therapy of RSV + EZ leads to significant lipid-lowering effects in the short term and long term, with the most notable reductions in LDL-C and TC, though HDL-C tends to decrease, particularly in the short-term and long-term groups.

### Safety of rosuvastatin versus rosuvastatin plus ezetimibe

#### Liver enzyme and gastrointestinal symptoms

**Fig. 4:**
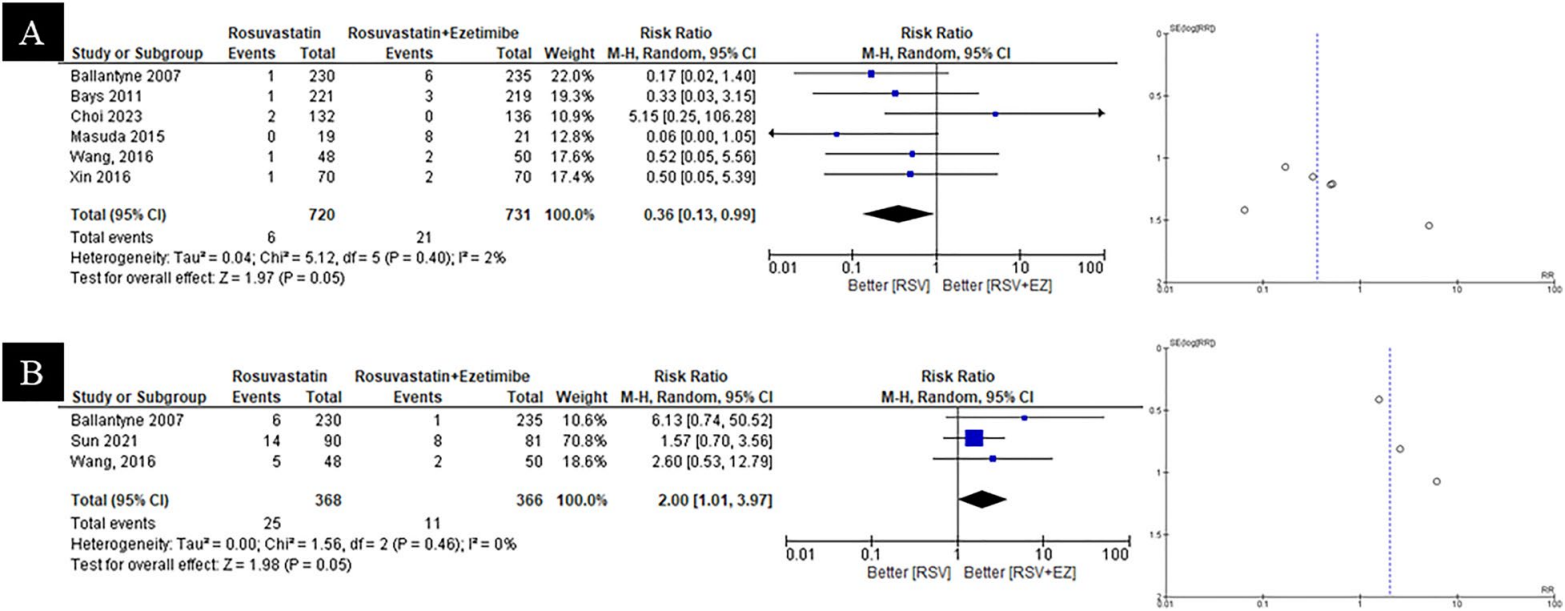
Safety outcome of RSV vs RSV plus ezetimibe for **A** liver enzyme, **B** gastrointestinal symptoms

Relative to RSV monotherapy, the combination of RSV + EZ demonstrated varied effects on patient outcomes, as illustrated in Fig. 4A and 4B. Figure 4A presents the findings from six studies assessing the safety of RSV + EZ compared to RSV alone, indicating that RSV monotherapy significantly increased the risk of elevated liver enzymes compared to RSV + EZ (RR = 0.36; 95% CI = 0.13–0.99; *p* = 0.05). Conversely, Fig. 4B shows data from three studies regarding specific adverse effects (gastrointestinal symptoms), with a pooled RR of 2.00 (95% CI: 1.01—3.97; *p* = 0.05; I2 = 0%). This indicates that the RSV + EZ combination significantly increased the risk of gastrointestinal symptoms compared to RSV monotherapy.

#### Myalgia and Angina Pectoris

**Fig. 5:**
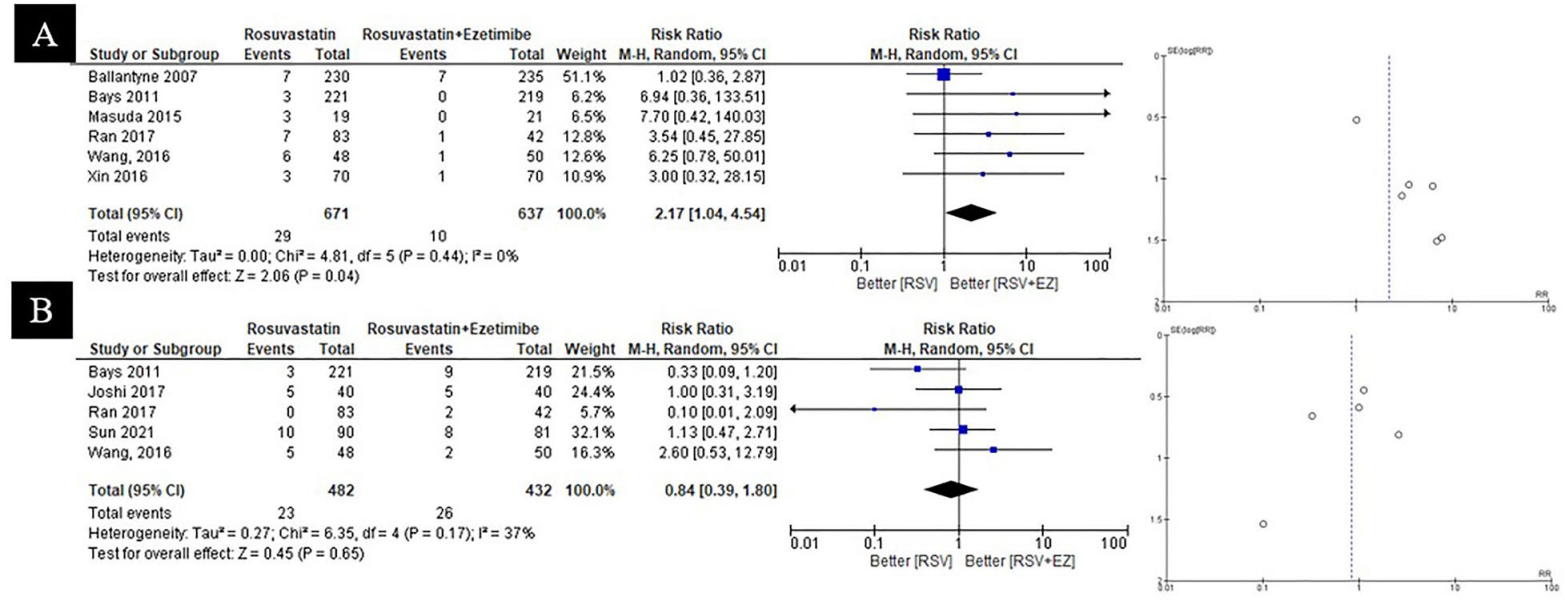
Safety outcome of RSV vs RSV plus ezetimibe for **A** myalgia, **B** angina pectoris

Results from seven studies comparing RSV monotherapy to the combination of RSV + EZ revealed varied safety outcomes regarding myalgia and angina pectoris. As shown in Fig. 5A, RSV + EZ significantly increased the risk of myalgia compared to RSV alone (RR = 2.17; 95% CI = 1.04–4.54; *p* = 0.04). In contrast, Fig. 5B indicate that RSV + EZ did not significantly affect the occurrence of angina pectoris events when compared to RSV monotherapy (RR = 0.84; 95% CI = 0.39–1.80; p = 0.65). Overall, while combination therapy may elevate the risk of myalgia, it does not significantly impact the incidence of angina pectoris relative to statin monotherapy.

## Discussion

Combination therapy of RSV, aHMG-CoA reductase inhibitor, and EZ, a cholesterol absorption inhibitor, has shown notable improvements in lipid profile management among patients with high-risk CAD. The combination of RSV and EZ demonstrated significant reductions in key lipid markers when compared to RSV monotherapy. Our findings indicate that the combination of RSV and EZ significantly improves lipid profiles in patients with CAD compared to RSV monotherapy (Fig. 3). This outcome is further supported by evidence from the EXPLORER and NSTE-CAD trials, which demonstrated that the RSV + EZ combination significantly reduced LDL-C levels more effectively than RSV alone, enabling more patients to achieve LDL-C goals. These findings underscore the enhanced efficacy of combination therapy in lipid management. [1, 4] Numerous studies have highlighted the high prevalence of lipid abnormalities in the general population, which constitutes a significant risk factor for CVDs. [19] If the economic burden of CVDs is to be reduced, LDL-C levels must be decreased to recommended targets. Unfortunately, a substantial proportion of patients, particularly those at high or very high risk, fail to achieve their LDL-C objectives, often due to inadequate escalation of lipid-lowering therapy. [20] The relationship between CAD and LDL-C levels has been extensively documented. [21] Recent research has indicated that reducing LDL-C levels below current targets is beneficial for high-risk individuals, with larger reductions correlating with improved clinical outcomes. Additionally, it has been demonstrated that statins can either accelerate or hinder the course of coronary atherosclerosis. [3]

Ezetimibe lowers cholesterol absorption by focusing on LDL-C uptake at the jejunal enterocyte brush border, whereas statins block the enzyme HMG-CoA reductase. Twenty-six statins and ezetimibe work in distinct ways to lower cholesterol. The mechanisms by which these two drugs operate are different. The combination of rosuvastatin with ezetimibe has proven to be more effective and safe than statin monotherapy in a number of patient populations, resulting in higher reductions of LDL-C and increases of HDL-C, as well as positive pleiotropic effects. According to worldwide guidelines, ezetimibe should be used as an adjuvant to statins in individuals who are unable to achieve LDL-C objectives despite taking the highest dose of statins that they can tolerate. [22] The study's main conclusion is that, in comparison with statin monotherapy, rosuvastatin and ezetimibe together significantly improve lipid profiles in CAD patients. These findings are consistent with the IMPROVE-IT research, which discovered that ezetimibe plus statins reduced LDL-C and atherosclerotic vascular event recurrences more effectively than statin monotherapy without worsening side effects. [23]

Rosuvastatin has distinct pharmacologic and pharmacokinetic properties, including as strong reduction of LDL-C, low CYP3 A4 interaction potential, and restricted penetration of extrahepatic tissue. [24] It is a fully synthetic HMG-CoA reductase inhibitor, whereas other statins are derived from natural mevinic or synthetic heptenoic acid precursors. [4] Other HMG-CoA reductase inhibitors are derived from synthetic heptenoic acid (atorvastatin, fluvastatin) or natural mevinic acid (lovastatin, simvastatin, pravastatin). [25] By binding to the Niemann–Pick C1-Like 1 (NPC1L1) protein, ezetimibe, a new cholesterol absorption inhibitor, decreases the absorption of dietary and biliary cholesterol in the small intestine. This process causes hepatic LDL-C receptors to be upregulated and improves the removal of circulating LDL-C [3]. Ezetimibe and rosuvastatin together dramatically enhanced the lipid profile, resulting in a 50% decrease in LDL-C levels, according to the I-ROSETTE research. Because the two drugs use different metabolic pathways, this combination therapy was also more well tolerated than an equivalent amount of rosuvastatin alone [26]. International studies have demonstrated that adding ezetimibe to statins further lowers LDL-C levels by 14–25%, independent of previous statin therapy, while doubling the statin dosage only results in an additional 6–7% reduction in LDL-C [4]. The current study demonstrated the effectiveness of rosuvastatin and ezetimibe combination therapy in complete lipid control by achieving significant reductions in all lipid profiles, including total cholesterol, LDL-C, HDL-C, and triglycerides.

Statins are primarily metabolized by hepatic cytochrome P450 enzymes, particularly CYP3 A4 and CYP3 A5, which include agents such as atorvastatin and simvastatin. A substantial number of other drugs are also processed by these enzymes, which can exacerbate drug-related side effects through metabolic interactions. In contrast, rosuvastatin is not significantly metabolized by CYP3 A4; instead, it is predominantly excreted in an unmetabolized form via the bile into feces. Partial metabolism by CYP2 C9 further reduces the likelihood of drug–drug interactions with rosuvastatin. Ezetimibe also minimizes such interactions by inhibiting intestinal cholesterol absorption and avoiding cytochrome P450 metabolism altogether. [4] Rosuvastatin and ezetimibe have complementary modes of action, which improves their ability to lower LDL-C. [20] For those who are unable to reach LDL-C targets with the maximum tolerable intensity of statin monotherapy, this combination has shown significant efficacy in lowering LDL-C in patients at risk of or with established CVD. [8] Nevertheless, research is still being done to determine the best dosage ratio and which statin is best for combination therapy with EZ. According to preliminary research, ezetimibe (10 mg) and rosuvastatin (5–10 mg) at lower dosages can have lipid-lowering effects that are on par with those of rosuvastatin alone (e.g., 20 mg). When compared to higher-dose statin monotherapy, this method has been linked to a lower incidence of side effects, including symptoms related to the muscles. For example, a research comparing RSV 5 mg/EZ 10 mg with RSV 20 mg revealed that while the combination medication group saw fewer side events, their LDL-C levels decreased similarly [27]. Additionally, a meta-analysis demonstrated that RSV 10 mg/EZ 10 mg was superior to RSV 20 mg in achieving LDL-C targets and provided improved safety outcomes [26]. The clinical safety of this combination medication has to be further investigated, especially with regard to its long-term effects on serious adverse cardiovascular events. To more clearly characterize the therapeutic role and safety profile, studies that concentrate on clinical outcomes are crucial. Overall, the results of this study (Figs. 4–5) show that rosuvastatin monotherapy was linked to a higher, albeit non-significant, risk of raised liver enzyme levels, pointing to a possible safety benefit of rosuvastatin and ezetimibe combination therapy. This finding is corroborated by a study by Kim et al. (2018), which showed that the combination therapy reduced LDL-C levels considerably more than rosuvastatin monotherapy while maintaining a similar safety profile in terms of side effects related to the liver and muscles [28]. However, the combined therapy was linked to a marginally non-significant increase in myalgia and a slightly higher risk of gastrointestinal problems, which may have an impact on patient tolerance. Higher-intensity statin therapy was linked to a higher incidence of muscle-related complaints, including myalgia, according to a network meta-analysis that included 153,000 patients. The extent of this risk varied depending on the patient's characteristics and dosage. [29]

**Fig. 6:**
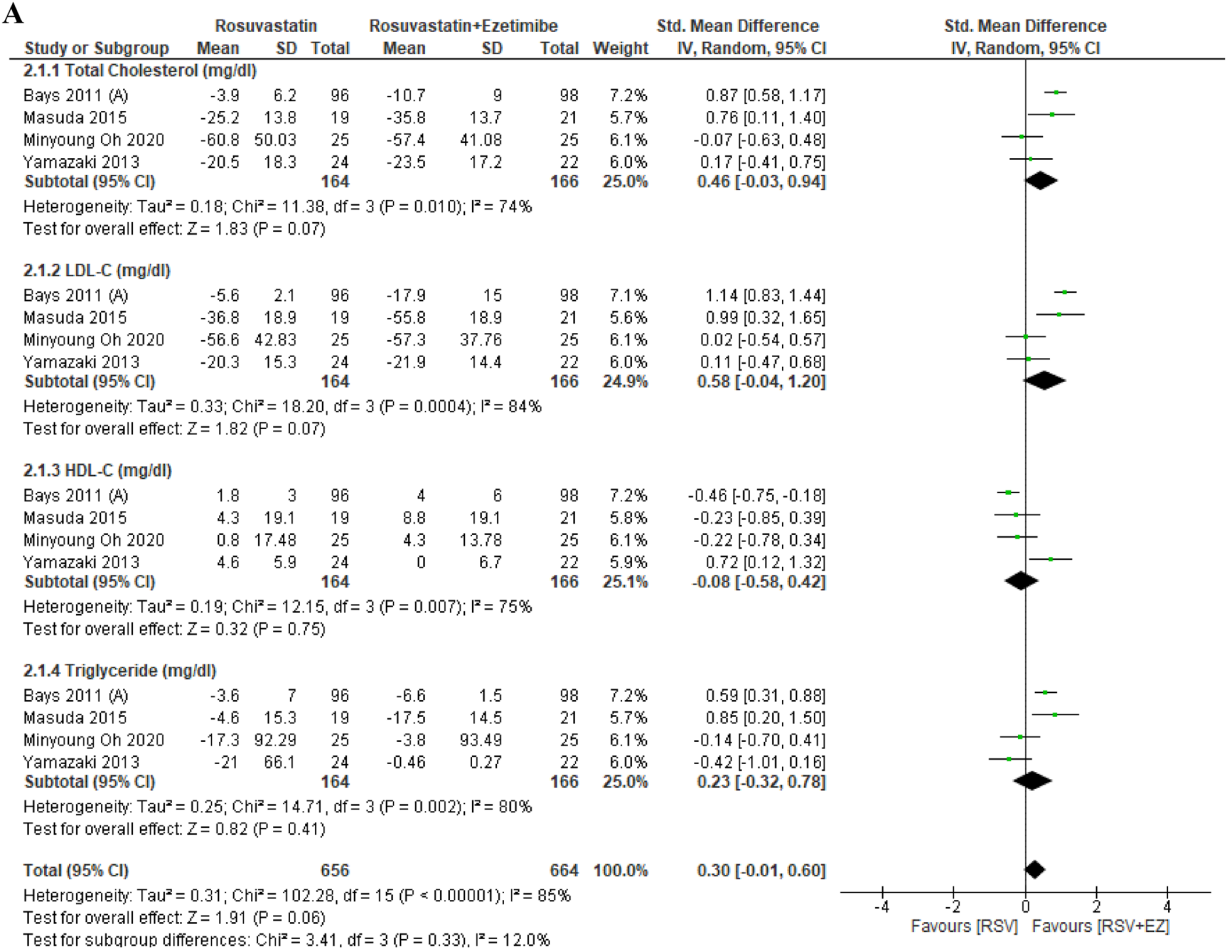

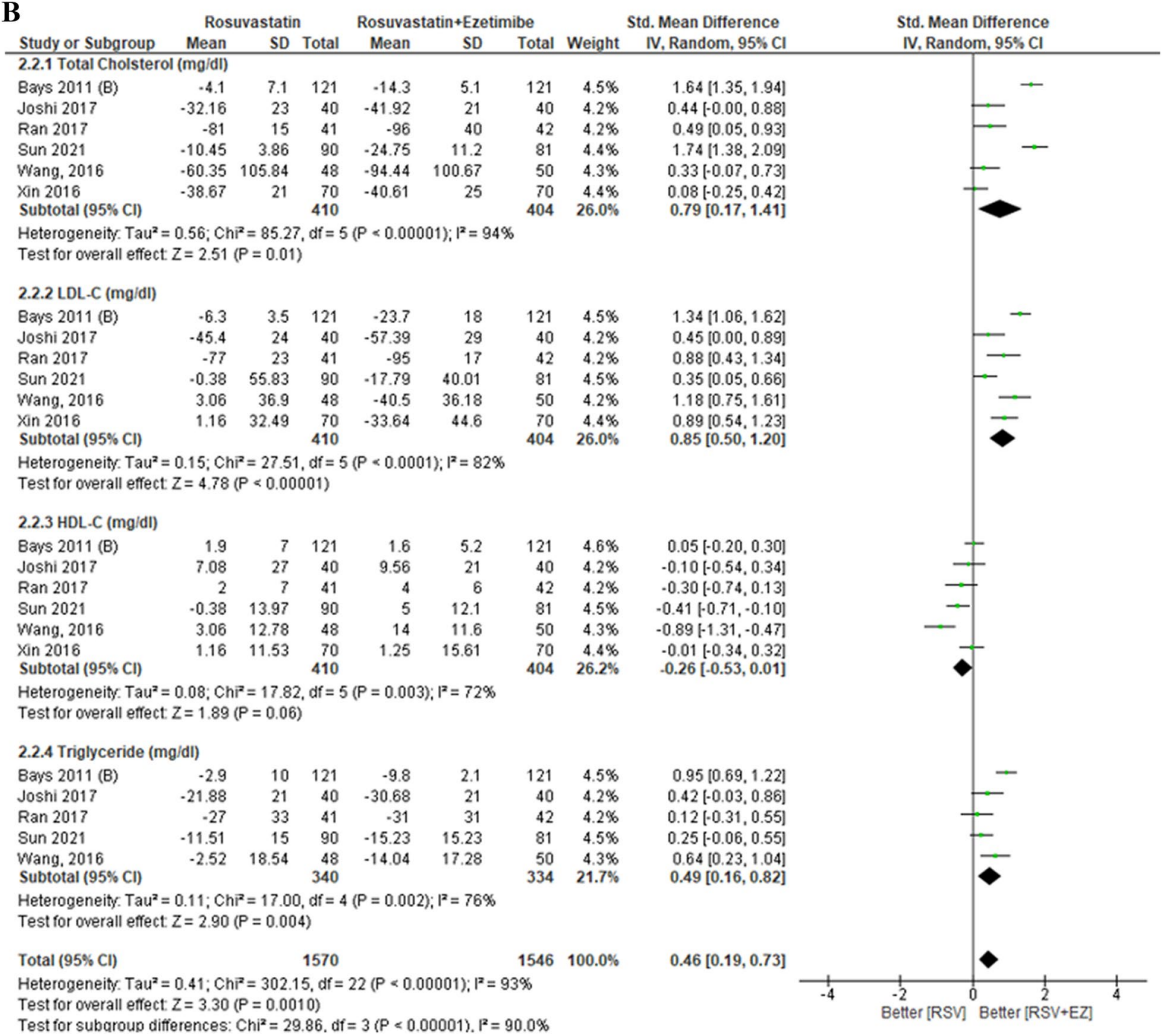

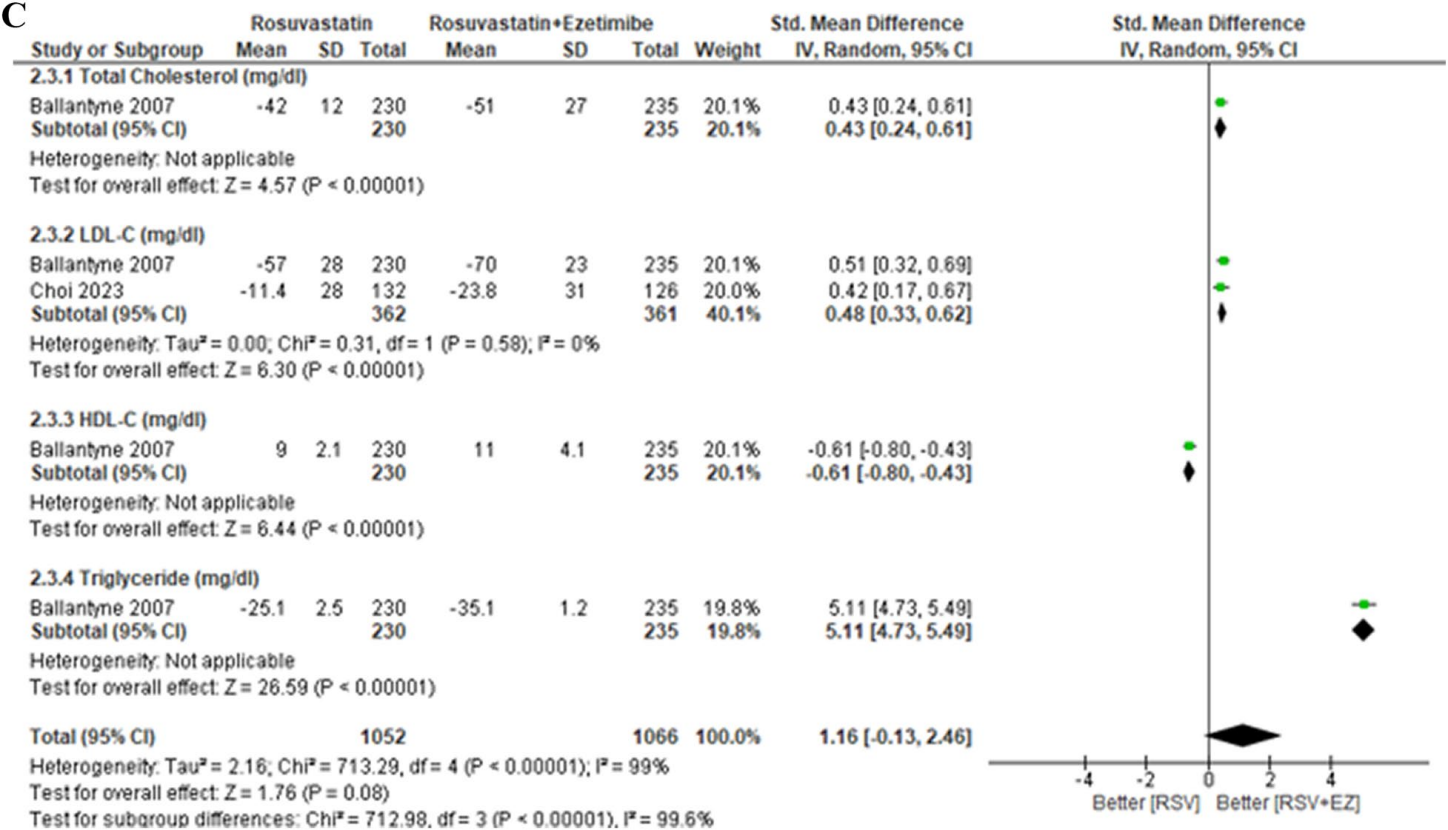
Lipid profile subgroup analysis by dose intensity. **A** Low-Dose RSV (RSV/EZ 2.5–5 mg/10 mg vs. RSV 5–10 mg), **B** Moderate-Dose RSV (RSV/EZ 10 mg/10 mg vs. RSV 10 mg), and **C** High-Dose RSV (RSV/EZ 20–40 mg/10 mg vs. RSV 20–40 mg)

In the lipid profile subgroup analysis by dose intensity (Fig. 6A-C), the results indicated a clear dose-dependent effect on lipid parameters. In the low-dose group (RSV/EZ 2.5–5 mg/10 mg vs RSV 5–10 mg), significant improvements were observed in total cholesterol (SMD 0.46, *p* = 0.007) and LDL-C (SMD 0.58, *p* = 0.007), but HDL-C showed no significant improvement (SMD − 0.08, p = 0.75), and triglycerides (TG) had a modest but non-significant reduction (SMD 0.23, *p* = 0.41). In the moderate-dose group (RSV/EZ 10 mg/10 mg vs RSV 10 mg), more substantial reductions in TC (SMD 0.79, *p* < 0.00001), LDL-C (SMD 0.85, *p* < 0.00001), and triglycerides (SMD 0.49, *p* = 0.004) were observed, with a slight increase in HDL-C (SMD −0.26, *p* = 0.003). For the high-dose group (RSV/EZ 20–40 mg/10 mg vs RSV 20–40 mg), TC (SMD 0.43, *p* < 0.00001) and LDL-C (SMD 0.48, *p* < 0.00001) reductions were significant, along with a marked reduction in triglycerides (SMD 5.11, *p* < 0.00001). However, HDL-C showed a significant increase (SMD − 0.61, *p* < 0.00001). These findings support earlier studies such as Ballantyne et al. (2007), which demonstrated that higher doses of combination therapy (rosuvastatin and ezetimibe) result in more significant LDL-C reductions [1]. Similarly, studies by Lorenzi et al. (2018) and Vaverkova et al. (2011) highlighted that rosuvastatin with ezetimibe therapy significantly reduced LDL-C levels and improved lipid profiles. [30, 31]

**Fig. 7:**
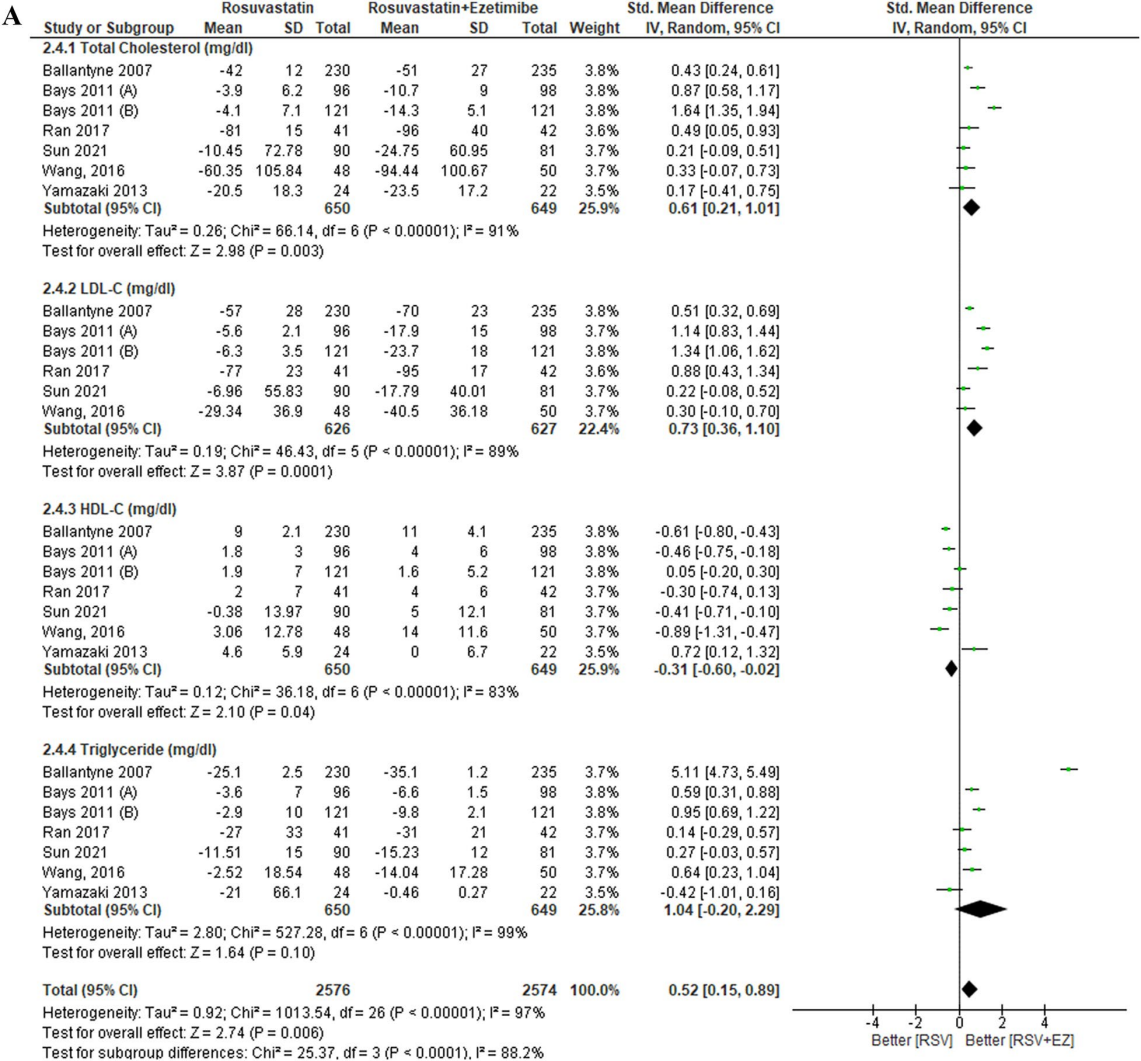

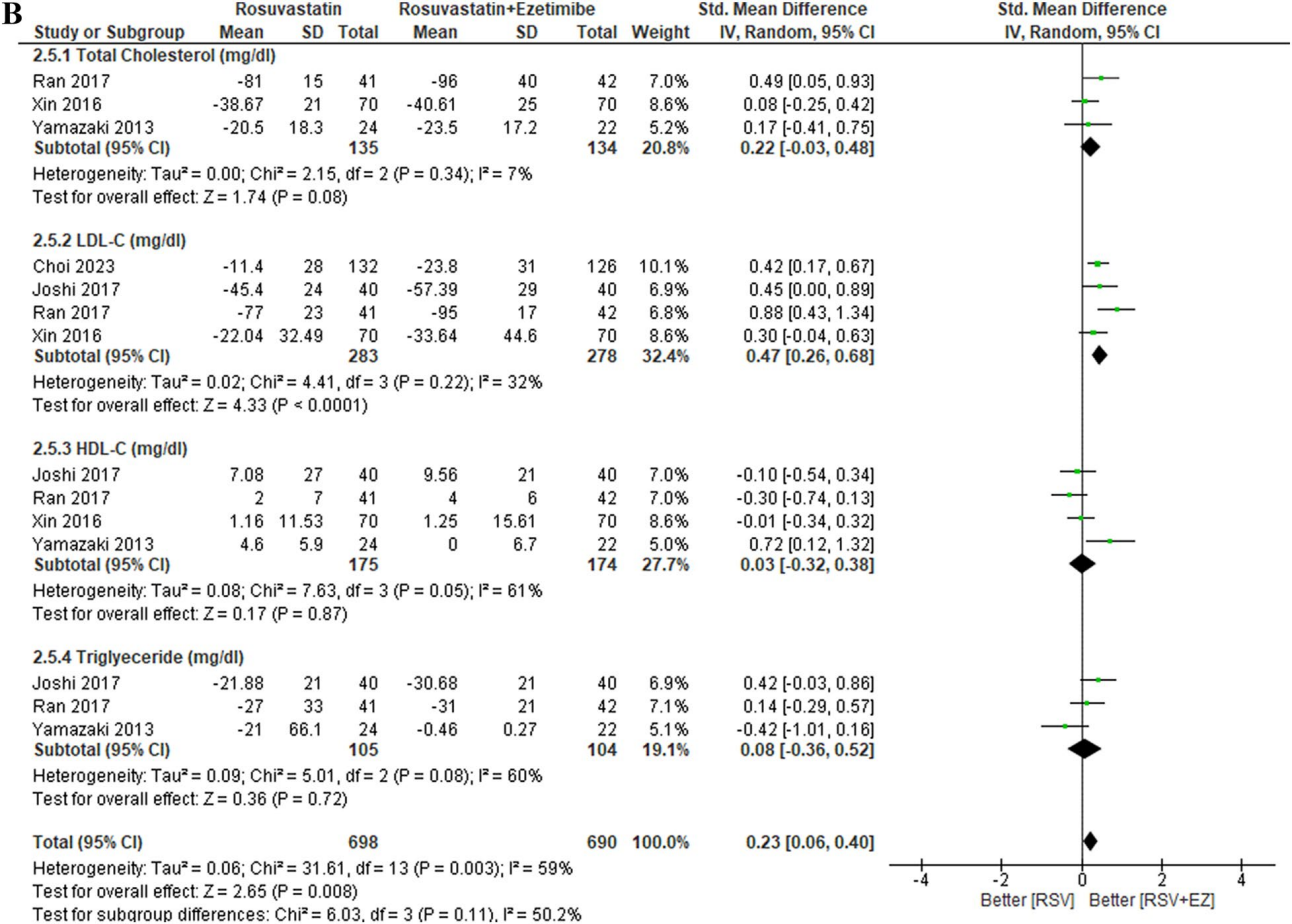

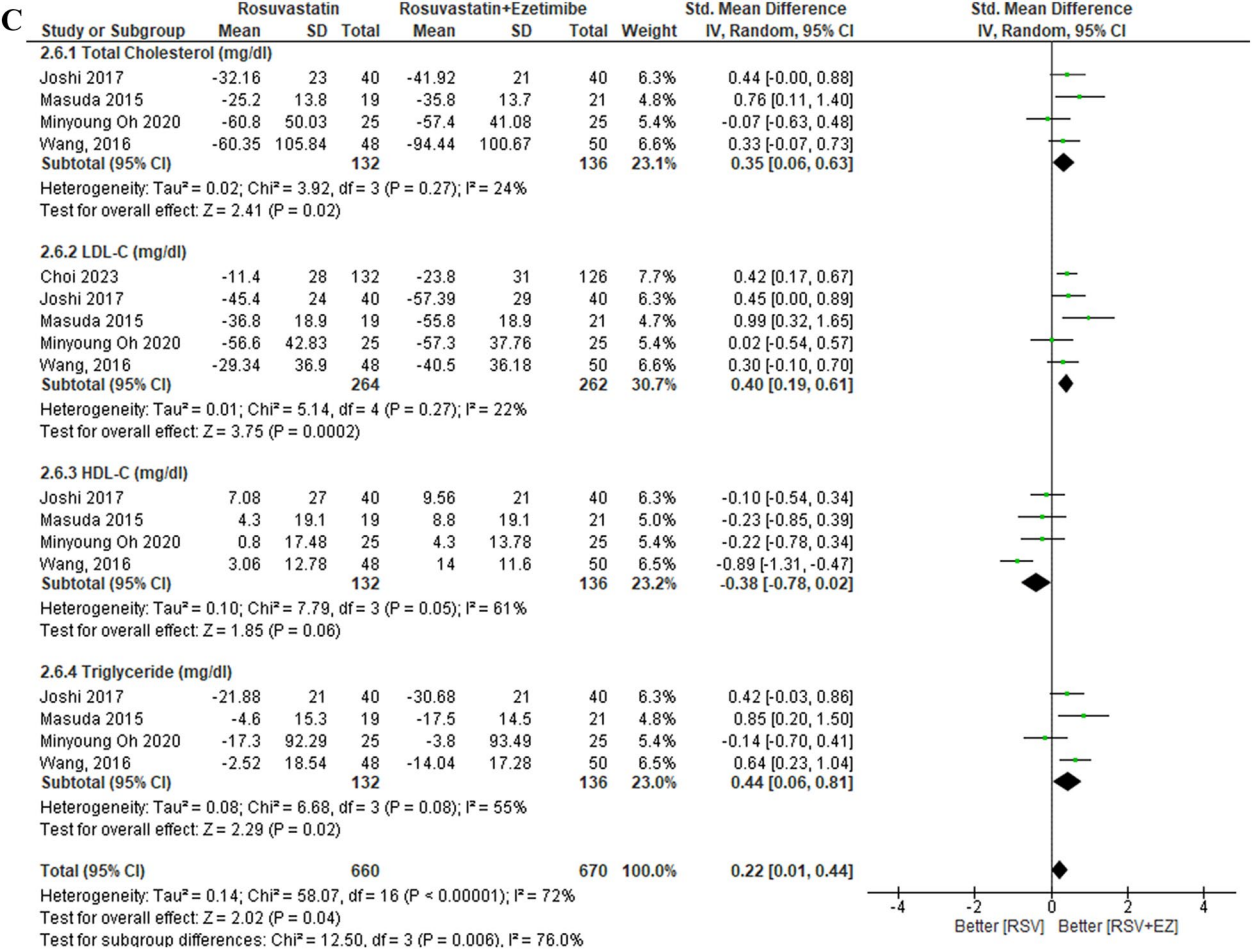
Lipid profile subgroup analysis by treatment duration. (A) Short-Term (<6 weeks), (B) Mid-Term (12 weeks), and (C) Long-Term (> 12 weeks)

In addition to the dose intensity, the treatment duration (Fig. 7A-C) also played a critical role in determining the effectiveness of rosuvastatin plus ezetimibe (RSV + EZ). In the short-term group (≤ 6 weeks), there were significant improvements in TC (SMD 0.61, *p* = 0.003) and LDL-C (SMD 0.73, *p* = 0.0001), with HDL-C showing a slight increase (SMD − 0.31, *p* = 0.004). However, triglycerides did not show a significant reduction (SMD 1.04, *p* = 0.10). In the mid-term group (12 weeks), LDL-C showed significant reductions (SMD 0.47, *p* < 0.0001), but TC improvements were modest (SMD 0.22, p = 0.08), and no significant changes in HDL-C or TG were observed. In the long-term group (> 12 weeks), TC (SMD 0.35, *p* = 0.002) and LDL-C (SMD 0.40, *p* = 0.0002) continued to improve, while HDL-C showed a slight increase (SMD 0.38, *p* = 0.06), and triglycerides improved significantly (SMD 0.44, *p* = 0.02). These results align with studies by Ballantyne et al. (2007), who demonstrated superior lipid-lowering effects of combination therapy in the long term compared to monotherapy [1]. The study by Lorenzi et al. (2018) also emphasized the long-term benefits of combining statins with ezetimibe in reducing lipid levels, particularly in patients with high LDL-C [30]. A study by Vaverkova et al. (2011) observed similar benefits in lipid reduction in longer durations, supporting the sustained efficacy of combination therapy. [31]

Although ezetimibe, when added to statin therapy, effectively lowers LDL cholesterol, gastrointestinal side effects, including abdominal discomfort, have been documented [25]. Despite these differences, no significant variation in the incidence of angina pectoris events was observed between rosuvastatin monotherapy and combination therapy, suggesting comparable efficacy in reducing ischemic events. These findings underscore the importance of balancing efficacy and safety when choosing between rosuvastatin monotherapy and its combination with ezetimibe for managing lipid profiles in patients with coronary artery disease. The data further highlight the necessity of personalized treatment strategies to optimize lipid-lowering therapy while monitoring for potential adverse effects, including liver enzyme elevations, myalgia, gastrointestinal symptoms, and angina pectoris. Supporting this, a meta-analysis by Dadzie et al. (2024) demonstrated that the combination significantly improved lipid parameters, including LDL-C, total cholesterol, and triglycerides, compared to rosuvastatin alone in patients with type 2 diabetes, though it showed slightly less impact on glycemic control [32]. Similarly, Kang et al. (2024) found that a low-dose rosuvastatin (5 mg) combined with ezetimibe (10 mg) produced LDL-C reductions comparable to those achieved with high-dose rosuvastatin monotherapy (20 mg), with slightly better results in total cholesterol lowering [26]. Kim et al. (2018) further confirmed that combination therapy not only helped more patients reach LDL-C targets than monotherapy but also maintained improvements over time [28]. These studies suggest that different dosing strategies—either increasing rosuvastatin or adding ezetimibe—can achieve similar lipid-lowering efficacy, though the combination approach may allow effective management at lower statin doses, potentially reducing statin-associated side effects.

This research is limited by the heterogeneity of included trials in terms of patient populations, treatment durations, and dosing regimens. Additionally, the relatively small number of high-quality randomized controlled trials and the lack of long-term outcome data may affect the generalizability of the findings. Potential publication bias and inconsistent reporting of adverse events across studies may also influence the safety assessment.

## Conclusion

The results suggest that combining RSV plus EZ effectively enhances the lipid profile in patients with high-risk CAD, resulting in decreased levels of total cholesterol, LDL-C, and triglycerides. RSV monotherapy was associated with increased HDL-C compared to the combination therapy of RSV + EZ; however, RSV monotherapy was associated with a greater risk of elevated liver enzymes, whereas RSV + EZ therapy was linked to an increased risk of gastrointestinal symptoms and myalgia. Furthermore, RSV + EZ did not significantly affect the incidence of angina pectoris. In conclusion, RSV + EZ may provide notable lipid-lowering benefits for high-risk CAD patients, but the potential risks, particularly liver enzyme, gastrointestinal symptoms and myalgia, should be carefully monitored and managed.

## Supplementary Information

Additional file 1.

## Data Availability

No datasets were generated or analysed during the current study.

## Abbreviations

CAD: Coronary artery disease
CHD: Coronary heart disease
CI: Confidence interval
CVD: Cardiovascular disease
CYP2 C9: Cytochrome P450 2C9
CYP3 A4: Cytochrome P450 3A4
CYP3 A5: Cytochrome P450 3A5
EZ: Ezetimibe
HDL-C: High-density lipoprotein cholesterol
HMG-CoA: Hydroxymethylglutaryl-coenzyme A
IMPROVE-IT: Improved reduction of outcomes: vytorin efficacy international trial
LDL-C: Low-density lipoprotein cholesterol
NPC1L1: Niemann–Pick C1-Like 1
NSTE-CAD: Non-ST-elevation acute coronary syndrome
PRISMA: Preferred reporting items for systematic reviews and meta-analyses
PROSPERO: International prospective register of systematic reviews
RCT: Randomized controlled trial
RoB: Risk of bias
RSV: Rosuvastatin
SGOT: Serum glutamic-oxaloacetic transaminase (AST)
SGPT: Serum glutamic-pyruvic transaminase (ALT)
T2DM: Type 2 diabetes mellitus
TG: Triglycerides

## References

[1] Ballantyne CM, Weiss R, Moccetti T et al (2007) Efficacy and safety of rosuvastatin 40 mg alone or in combination with ezetimibe in patients at high risk of cardiovascular disease (results from the EXPLORER study). Am J Cardiol 99(5):673–680. 10.1016/j.amjcard.2006.10.022 17317370 10.1016/j.amjcard.2006.10.022

[2] Wang X, Zhao X, Li L, Yao H, Jiang Y, Zhang JY (2016) Effects of combination of ezetimibe and rosuvastatin on coronary artery plaque in patients with coronary heart disease. Heart Lung Circ 25(5):459–465 26687339 10.1016/j.hlc.2015.10.012

[3] Masuda J, Tanigawa T, Yamada T et al (2015) Effect of Combination Therapy of Ezetimibe and Rosuvastatin on Regression of Coronary Atherosclerosis in Patients With coronary artery disease. Int Heart J 56:278–285 25902885 10.1536/ihj.14-311

[4] Ran D, Nie H, juan, Gao Y lin, et al (2017) A randomized, controlled comparison of different intensive lipid-lowering therapies in Chinese patients with non-ST-elevation acute coronary syndrome (NSTE-ACS): Ezetimibe and rosuvastatin versus high-dose rosuvastatin. Int J Cardiol 235:49–55. 10.1016/j.ijcard.2017.02.099 28291622 10.1016/j.ijcard.2017.02.099

[5] Sun C, Zheng W, Liang L, Liu Z, Sun W, Tang R (2021) Ezetimibe improves rosuvastatin effects on inflammation and vascular endothelial function in acute coronary syndrome patients undergoing PCI. J Interv Cardiol 2021:1–7. 10.1155/2021/2995602 10.1155/2021/2995602 PMC8443370 34566523

[6] Lewek J, Niedziela JT, Desperak P et al (2023) Intensive statin therapy versus upfront combination therapy of statin and ezetimibe in patients with acute coronary syndrome: a propensity score matching analysis based on the PL-ACS Data. J Am Heart Assoc Cardiovasc and Cerebrovasc Disease. 10.1161/JAHA.123.030414 10.1161/JAHA.123.030414 PMC10547305 37671618

[7] Yang YJ, Lee SH, Kim BS et al (2017) Combination therapy of rosuvastatin and ezetimibe in patients with high cardiovascular risk. Clin Ther 39(1):107–117 28007331 10.1016/j.clinthera.2016.11.014

[8] Lee J, Hwang YC, Lee WJ et al (2020) Comparison of the efficacy and safety of rosuvastatin/ezetimibe combination therapy and rosuvastatin monotherapy on lipoprotein in patients with type 2 diabetes: multicenter randomized controlled study. Diabetes Therapy 11:859–871 32065359 10.1007/s13300-020-00778-1 PMC7136381

[9] Wu Y, Chen K, Yang SY, Fu X, Ming JZ (2014) Effects of the combination of ezetimibe and rosuvastatin on coronary atherosclerotic plaque. Central Plains Med J 41:57–59

[10] Cannon CP, Blazing MA, Giugliano RP et al (2015) Ezetimibe added to statin therapy after acute coronary syndromes. N Engl J Med 372(25):2387–2397 26039521 10.1056/NEJMoa1410489

[11] Stein EA, Ose L, Retterstøl K et al (2007) Further reduction of low-density lipoprotein cholesterol and C-reactive protein with the addition of ezetimibe to maximum-dose rosuvastatin in patients with severe hypercholesterolemia. J Clin Lipidol 1(4):280–286 21291692 10.1016/j.jacl.2007.07.003

[12] Chang SH, Wu LS, Lee CH et al (2015) Simvastatin-ezetimibe combination therapy is associated with a lower rate of major adverse cardiac events in type 2 diabetics than high potency statins alone: A population-based dynamic cohort study. Int J Cardiol 190:20–25 25912112 10.1016/j.ijcard.2015.04.121

[13] Namal E, Sener N, Ulaş T, Akçalı Z, Oztekin E, Borlu F (2011) Effects of different statins, ezetimibe/simvastatin combination on hsCRP levels in unstable angina pectoris and non-ST elevation myocardial infarction patients: a randomized trial. Anadolu Kardiyol Derg 11(8):703–710 22088858 10.5152/akd.2011.192

[14] Deharo P, Pankert M, Quilici J et al (2014) Safety and effectiveness of the association ezetimibe-statin (E-S) versus high dose rosuvastatin after acute coronary syndrome: the SAFE-ES study. Ann Cardiol Angeiol (Paris) 63(4):222–227 24861503 10.1016/j.ancard.2014.04.018

[15] Hong SJ, Jeong HS, Ahn JC, Phase A III et al (2018) Multicenter, randomized, double-blind, active comparator clinical trial to compare the efficacy and safety of combination therapy with ezetimibe and rosuvastatin versus rosuvastatin monotherapy in patients with hypercholesterolemia: I-ROSETTE (ildong rosuvastatin & ezetimibe for hyperch. Clin Ther 40(2):226–241 29402522 10.1016/j.clinthera.2017.12.018

[16] Cannon CP, Giugliano RP, Blazing MA et al (2008) Rationale and design of IMPROVE-IT (IMProved Reduction of Outcomes: Vytorin Efficacy International Trial): comparison of ezetimbe/simvastatin versus simvastatin monotherapy on cardiovascular outcomes in patients with acute coronary syndromes. Am Heart J 156(5):826–832 19061694 10.1016/j.ahj.2008.07.023

[17] Page MJ, McKenzie JE, Bossuyt PM et al (2020) The PRISMA statement: An updated guideline for reporting systematic reviews. The BMJ 2021:372. 10.1136/bmj.n71 10.1136/bmj.n71 PMC8005924 33782057

[18] The Cochrane Collaboration., Review manager (RevMan) Version 5.4. 2020. Accessed April 13, 2024. https://training.cochrane.org/System/Files/Uploads/Protected_file/RevMan5.4_user_guide.Pdf

[19] Setny M, Jankowski P, Krzykwa A et al (2021) Management of dyslipidemia in women and men with coronary heart disease: results from polaspire study. J Clin Med. 10.3390/jcm10122594 34208351 10.3390/jcm10122594 PMC8231115

[20] Barrios V, Escobar C (2021) Fixed-dose combination of rosuvastatin and ezetimibe: treating hypercholesteremia according to cardiovascular risk. Expert Rev Clin Pharmacol. 10.1080/17512433.2021.1925539 33970743 10.1080/17512433.2021.1925539

[21] Yamazaki D, Ishida M, Watanabe H et al (2013) Comparison of anti-inflammatory effects and high-density lipoprotein cholesterol levels between therapy with quadruple-dose rosuvastatin and rosuvastatin combined with ezetimibe. Lipids Health Dis. 10.1186/1476-511X-12-9 23374898 10.1186/1476-511X-12-9 PMC3598241

[22] Chilbert MR, Vanduyn D, Salah S, Clark CM, Ma Q (2022) Combination therapy of ezetimibe and rosuvastatin for dyslipidemia: current insights. Drug Des Devel Ther. 10.2147/DDDT.S332352 35832642 10.2147/DDDT.S332352 PMC9273150

[23] Mostaza JM, Escobar C (2024) Rosuvastatin-based lipid-lowering therapy for the control of LDL cholesterol in patients at high vascular risk. J Clin Med. 10.3390/jcm13071894 38610659 10.3390/jcm13071894 PMC11012264

[24] Luvai A, Mbagaya W, Hall AS, Barth JH (2012) Rosuvastatin: A review of the pharmacology and clinical effectiveness in cardiovascular disease. Clin Med Insights Cardiol. 10.4137/CMC.S4324 22442638 10.4137/CMC.S4324 PMC3303484

[25] Gagné C, Bays HE, Weiss SR et al (2002) Efficacy and safety of <em>ezetimibe</em> added to ongoing statin therapy for treatment of patients with primary hypercholesterolemia. Am J Cardiol 90(10):1084–1091. 10.1016/S0002-9149(02)02774-1 12423708 10.1016/s0002-9149(02)02774-1

[26] Kang Y, Park JM, Lee SH (2024) Moderate-intensity rosuvastatin/ezetimibe combination versus quadruple-dose rosuvastatin monotherapy: a meta-analysis and systemic review. Yonsei Med J 65(1):19–26. 10.3349/ymj.2023.0285 38154476 10.3349/ymj.2023.0285 PMC10774651

[27] Rhee MY, Kim KJ, Kim SH et al (2019) Ezetimibe and rosuvastatin combination treatment can reduce the dose of rosuvastatin without compromising its lipid-lowering efficacy. Clin Ther 41(12):2571–2592. 10.1016/j.clinthera.2019.10.010 31727361 10.1016/j.clinthera.2019.10.010

[28] Kim W, Yoon YE, Shin SH et al (2018) Efficacy and safety of ezetimibe and rosuvastatin combination therapy versus those of rosuvastatin monotherapy in patients with primary hypercholesterolemia. Clin Ther 40(6):993–1013. 10.1016/j.clinthera.2018.04.015 29857919 10.1016/j.clinthera.2018.04.015

[29] Davis JW, Weller SC (2021) Intensity of statin therapy and muscle symptoms: a network meta-analysis of 153 000 patients. BMJ Open. 10.1136/bmjopen-2020-043714 34130955 10.1136/bmjopen-2020-043714 PMC8211057

[30] Lorenzi M, Ambegaonkar B, Baxter C, Jansen J, Zoratti M, Davies G (2018) Ezetimibe in high-risk, previously treated statin patients: a systematic review and network meta-analysis of lipid efficacy. Clin Res Cardiol 108:487–509. 10.1007/s00392-018-1379-z 30302558 10.1007/s00392-018-1379-z

[31] Averna M, Missault L, Vaverkova H et al (2011) Lipid-altering efficacy of switching to ezetimibe/simvastatin 10/20 mg versus rosuvastatin 10 mg in high-risk patients with and without metabolic syndrome. Diab Vasc Dis Res 8:262–270. 10.1177/1479164111418136 21859750 10.1177/1479164111418136

[32] Dadzie S, Tabowei G, Kaur M et al (2024) A comparison of rosuvastatin monotherapy and rosuvastatin plus ezetimibe combination therapy in patients with type 2 diabetes: a meta-analysis of randomized controlled trials. Cureus. 10.7759/cureus.61526 38957250 10.7759/cureus.61526 PMC11218846

