# Supplementary material for: Lipid profile and safety of rosuvastatin monotherapy versus rosuvastatin plus ezetimibe in high risk coronary artery disease: a systematic review and meta-analysis of randomized controlled trials
Source: Egypt Heart J. 2025 Jun 10;77:58. doi: 10.1186/s43044-025-00654-y (PMC12151963; doi:10.1186/s43044-025-00654-y)
Supplement: Supplementary file 1 — Additional file 1. [file 43044_2025_654_MOESM1_ESM.docx]

**Supplementary File**

**Table S1.** Search strategy used in each table

| Database | Keywords |
| --- | --- |
| Pubmed | #1 coronary heart disease [MeSH Terms] |
|  | #2 ((Coronary Heart Disease*[Title/Abstract] OR (“CHD” [Title/Abstract] OR (Coronary Artery Disease [Title/Abstract] OR (“CAD” [Title/Abstract])) |
|  | #3 #1 OR #2 |
|  | #4 Rosuvastatin [Supplementary Concept] |
|  | #5 ((Rosuvastatin [Title/Abstract] OR "High intensity lipid" [Title/Abstract] OR "Moderate High intensity lipid" [Title/Abstract] OR “RSV” [Title/Abstract])) |
|  | #6 #4 OR #5 |
|  | #7 Ezetimibe [Supplementary Concept] |
|  | #8 ((Ezetimibe [Title/Abstract] OR (“EZ” [Title/Abstract])) |
|  | #9 #7 OR #8 |
|  | #10 Rosuvastatin plus Ezetimibe [Supplementary Concept] |
|  | #11 ((Rosuvastatin plus Ezetimibe [Title/Abstract] OR “Rosuvastatin combination” [Title/Abstract])) |
|  | #12 #10 OR #11 |
|  | #13 #3 AND #6 AND #9 AND #12 |
| Science Direct | ("Acute Coronary Syndrome") AND ("Aspirin") OR ("Clopidogrel") OR ("Prasugrel") OR ("Ticagrelor") AND ("Aspirin Plus Clopidogrel") OR ("Aspirin Plus Ticagrelor") OR ("Aspirin Plus Prasugrel") |
| Google Scholar | ("ACS") OR ("Acute Coronary Syndrome") AND ("NSTE-ACS") OR ("Non-ST-elevation ACS") AND ("STE-ACS") OR ("ST-elevation ACS") AND ("CAD") OR ("Coronary Artery Disease") AND ("SAPT") OR ("Single Antiplatelet") OR ("Antiplatelet Monotherapy") OR ("Aspirin") OR ("Clopidogrel") OR ("Prasugrel") OR ("Ticagrelor") AND ("DAPT") OR ("Double Antiplatelet") OR ("Antiplatelet Combination") OR ("Aspirin Plus Clopidogrel") OR ("Aspirin Plus Ticagrelor") OR ("Aspirin Plus Prasugrel") OR ("Aspirin Plus P2Y12 Inhibitor") |
| Europe PMC | ("ACS" OR "Acute Coronary Syndrome" OR "NSTE-ACS" OR "Non-ST-elevation ACS" OR "STE-ACS" OR "ST-elevation ACS") AND ("CAD" OR "Coronary Artery Disease") AND ("SAPT" OR "Single Antiplatelet" OR "Antiplatelet Monotherapy" OR "Aspirin" OR "Clopidogrel" OR "Prasugrel" OR "Ticagrelor") AND ("DAPT" OR "Double Antiplatelet" OR "Antiplatelet Combination" OR "Aspirin Plus Clopidogrel" OR "Aspirin Plus Ticagrelor" OR "Aspirin Plus Prasugrel") |
